# Impact of media coverage on the transmission dynamics of TB with vaccines and treatment

**DOI:** 10.1371/journal.pone.0314324

**Published:** 2025-01-28

**Authors:** Kumneger Tadesse Mulugeta, Mohammed Yiha Dawed, Shewafera Wondimagegnhu Teklu

**Affiliations:** 1 Department of Mathematics, College of Natural and Computational Science, Debre Berhan University, Debre Berhan, Adis Ababa, Ethiopia; 2 Department of Mathematics, College of Natural and Computational Science, Hawassa University, Hawassa, Adis Ababa, Ethiopia; Kwame Nkrumah University of Science and Technology, GHANA

## Abstract

Tuberculosis (TB) is one of the deadly infectious diseases affecting millions of individuals throughout the world. The main objective of this study is to investigate the impact of media coverage on the transmission dynamics of TB with vaccine and treatment strategy using mathematical model analysis. In the qualitative analysis of the proposed model we proved the existence, uniqueness, positivity, and boundedness of the model solutions, investigated both the disease-free and endemic equilibrium points, computed the basic and effective reproduction numbers using next generation matrix approach, analyzed the stability analysis of the equilibrium points, the backward bifurcation using the Castillo-Chavez and Song theorem and we re-formulated the corresponding optimal control problem and analyzed by applying the Pontryagin’s Minimum Principle. In the model quantitative (numerical) analysis part, we performed the model parameters sensitivity analysis and carried out numerical simulation to verify the qualitative analysis results. The findings of the study indicate that if the reproduction number is less than one, the solution converges to the disease-free state, signifying the asymptotic stability of the TB-free steady state. Moreover, the existence of a backward bifurcation shows that the disease-free equilibrium coexists with one or more endemic equilibria, even when the basic reproduction number is less than 1. Furthermore, it is found that as media efficacy increases, the disease infection rate decreases, which consequently leads to an increase in prevention and treatment control strategies.

## 1 Introduction

Tuberculosis (TB) is a serious infectious disease caused by the bacterium Mycobacterium tuberculosis. This bacterium is an ancient bacterium that was proposed by Robert Koch as the cause of tuberculosis in 1882 [1]. It is a public health problem that spreads from person to person through the air when infected people sing, talk, cough, sneeze, or spit, and it remains inactive for years before becoming active. A quarter of the world’s population is estimated to be infected with TB disease [2]. The TB bacteria may exist in latent or active cases. This means that if someone has symptoms and feels pain, then the bacteria becomes active. But if someone has no symptoms and no feelings of sickness, then the bacteria is staying latent. TB is a preventable, treatable, and curable disease, and the major causes of TB morbidity and mortality are lack of access to early diagnosis, treatment, and prevention services and co-infection with other diseases. Diagnosis of latent TB infections and prompt treatment of active TB cases are important components of effective TB control [3]. TB has effective drug treatments, which were first developed in the 1940s. Bacille Calmette-Guérin (BCG) is the only licensed vaccine for the prevention of TB disease. It was developed almost 100 years ago, prevents severe forms of TB in children, and is widely used.

According to the World Health Organization, the cases of TB are still increasing. The WHO TB report of 2023 estimates 10.6 million people developed TB and caused 1.3 million deaths due to TB disease in 2022 [4]. Most of the people who fall ill with TB are living in low- and middle-income countries, and people in prison and mentally ill patients are also more likely to be at risk. This disease is the second-leading cause of death in the world from a single infectious disease after COVID-19, and its deaths are twice as high as HIV/AIDS [4]. Adults of their most productive age are more affected by the TB disease, with more cases in men than women. According to the study [5], the burdens of TB that challenge the health sector are: inadequate implementation of universal health coverage, insufficient coordination to address risk factors, and limited research and innovation. Wars and conflicts have their own negative effects in the community, to get early access social and healthcare services which resulting in devastating and long-lasting consequences. To eradicate TB from the society, the pivotal task is addressing the lack of social protection of TB. One of the actions to reduce the TB burden is to intensify research by considering different assumptions. When more research is done, more knowledge will be shared with the community, and people will become aware of TB.

Tuberculosis is highly infectious and still among the most common causes of death in the world. It is found in every country in the world and is responsible for economic devastation and the cycle of poverty and illness that binds communities and even entire countries. The COVID-19 pandemic has also set back TB control programs worldwide. Infection with HIV/AIDS increases the risk of developing M. tuberculosis infection and reactivation of latent TB infection by 5–15% [6]. The TB control program activities related to screening and diagnosis are challenged [7]. The study [8] also mentioned that latent tuberculosis infection is of great concern, especially in an aging population. Overall, ending the TB epidemic will require three rapid scientific advances: developing innovative diagnostic tools, the development and deployment of effective drugs to combat drug-susceptible and drug-resistant TB, and an effective TB vaccine [9]. Tuberculosis is categorized into two groups by anatomical site of disease: pulmonary (disease affecting the lung, which is the most common form of TB) and extra-pulmonary (disease affecting sites including lymph nodes).

Mass media is one of the important strategies for making changes in knowledge, attitudes, awareness, and opinions about TB and health-seeking behavior intentions for TB. Media coverage is a powerful tool for TB control and for influencing the community about the impact of TB. It also plays a pivotal role in mitigating a lack of awareness about TB disease. The researcher in the study [10] recommends that media campaigns have to be delivered for an unknown longer period of time until the changes in the perception of the risk of TB are embedded in the culture of the community. According to the research in [11], media awareness is important and effective tool to generate preventive control measures for communicable diseases. The study [12] also mentioned that the media coverage campaign for the vaccination with education campaign can decrease the infection in the community. The media is important to share problems between people and reduce the stigma against the disease. Media coverage including television, radio, newspapers, internet, and posters have an important role in preventing and controlling the epidemic disease [13].

Mathematical models have played a key role in the formulation of TB control strategies and the establishment of temporary goals for intervention programs [14]. Mathematical models can provide useful suggestions for the dynamics of TB transmission, particularly in relation to vaccine use and incidence rates [15]. Many researchers have performed mathematical modeling analyses on the dynamics of TB transmission regarding TB eradication and control [16]. Some of these, the researcher in [17] developed a mathematical model analysis on the dynamics of TB disease by considering two different treatment strategies, namely protective treatment for the latent TB-infected population and the main treatment applied to the infected populations. The study [18] developed a mathematical model analysis of TB by considering two classes of latently infected individuals with different exposures. The paper in [15] studies the transmission dynamics of TB by developing a mathematical model to analyze the impact of mixing proportional incidence rates with vaccination. The research on [11] developed a deterministic model for the transmission of TB, considering the impact of social media. An article in [19] attempted to describe and construct the epidemiology model of tuberculosis by including multidrug resistance. The researcher [20] developed a deterministic epidemic model to investigate the effect of treatment adherence and awareness on the dynamics of tuberculosis. The study in citemawira2020mathematical also developed the transmission dynamics of TB by considering the effects of hygiene consciousness as a control strategy against TB. The findings of the paper [3] show that infected populations are reduced when isolation and treatment rates and their effectiveness increase.

Many scholars have considered media coverage concept in their mathematical model construction to study the transmission dynamics of the diseases [10, 12]. The study in [21] attempted to formulate and analyze the transmission dynamics of TB with fast and slow progression, and media coverage of disease-related messages can influence the transmission rate to be reduced by a factor *e*^−*αM*^. The paper in [13] constructed a mathematical model to investigate the impact of media coverage on the spread and control of drug addiction by considering that media coverage decreases the contact rate by the factor $\beta e^{-m_{1}U-m_{2}H}$. The researcher in [12] developed a transmission mathematical model of the hepatitis *B* virus. In this study the transmission contact rate is reduced due to the media effect represented by the term like $\frac{\beta_{i}A}{\eta_{A}+A}$ where *i* = 1, 2 in the model. The media coverage may affect the incidence rate, and a nonlinear incidence rate can be approximated by various forms, such as in [22] *β*(*I*) = *μe*^−*mI*^ is the contact and transmission term, in [11] $\beta \left(I\right)=\left(\beta_{1}-\beta_{2}\frac{I}{m+I}\right)$ is the contact rate after media alert, and in [23] $\beta \left(I\right)=\left(1-M\right)\beta \frac{I}{N}$ where *M* is the sum of the media efficiency of information shared through Facebook, television, radio, and Twitter. The study [24, 25] explored mathematical model analysis of TB with a saturated incidence rate and a cost-effective analysis of multidrug-resistant TB in Ethiopia. In their work, they mentioned that the ministry of health must focus more on prevention strategies such as isolation of infectious people, early TB patient detection, distancing, treatment, and educational programs. Media has an important role in increasing human thinking skills and knowledge by transmitting various information and educational messages. To conduct educational programs or to make people aware of TB disease, media play a great role. In order to operationalize the media effect in this paper, we implement the media coverage in the force of infection as a function of $\beta_{2}\frac{I}{m+I}$ (Holling-type II functional response [26]), where *m* is media efficacy. As the efficiency of the media increases, the rate of spread of the disease may decrease.

The natural sciences (such as physics, biology, earth science, and chemistry), engineering disciplines (such as computer science and electrical engineering), and non-physical systems like the social sciences (such as economics, psychology, sociology, and political science) all use mathematical models. Computational mathematics is used to investigate the dynamics of cooperative occurrences in chemical reactions inside living organisms [27]. This research explore the dynamics of complex systems using mathematical models based on ordinary differential equations, paying special attention to chemical equilibrium and reaction speed. Further more, it emphasizes how well ordinary differential equations may represent the complicated system dynamics found in chemical reactions. A fractional differentiation combined with a fractal dimension mathematical model is used to analyse the dynamics of Ebola virus disease [28]. To reduce the continuous propagation of gonorrhea, the study [29] designed a mathematical model analysis by incorporating education, condom usage, vaccinations, and treatment as control strategies. As the researchers [30, 31] clarified, deterministic and fractal-fractional calculus mathematical models are important for the analysis of co-infection and disease dynamics, respectively. The study [32] also developed a mathematical model for the co-dynamics of diabetes and tuberculosis co-infection. Our study extends and improves the work [11] by incorporating the vaccine and treatment compartments in the model. The model also holds the reinfection of individuals who have recovered from TB infection and who have joined the treatment class of TB disease. Moreover, the model considers vaccines waning on the TB disease.

In this study we propose a mathematical model to understand the transmission dynamics of TB infection in a population. Our mathematical model is an extension of the work [11] which takes into account media coverage, Vaccination and the undergoing for treatment compartments. Additionally, we will introduce relapse behavior of the TB disease. Relapse can occur due to several factors, including incomplete treatment, the presence of drug-resistant strains, or re-infection with a new strain of the bacteria. Exogenous re-infection in TB transmission disease and imperfect vaccines are determines as common causes of the existence of backward bifurcation [33]. The backward bifurcation phenomenon in TB disease transmission models is that a stable endemic equilibrium coexists with a stable disease-free equilibrium when the associated reproduction number is less than unity, as has been observed in several transmission models. Thus, the backward bifurcation of our TB model has been presented and explained in Section 3. The optimal control problem is proposed by implementing prevention and treatment control strategies to reduce both the burden of disease and the cost of intervention strategies. In section 1, we write introduction, section 2 we formulate the mathematical model, section 3 we analyze the model, section 4 we extend the problem into optimal control, section 5 we do sensitivity analysis, in section 6 Numerical simulation will be done, and finally in section 7 we have recommendation and Conclusion.

## 2 The mathematical model formulation

**Fig 1:**
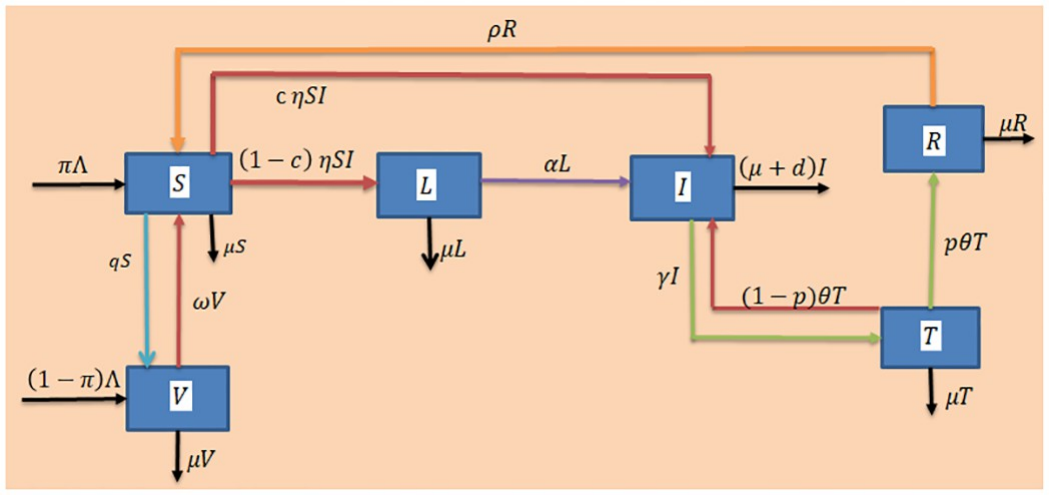
Flow chart of the mathematical model.

Media coverage plays role on human behavior and hence affects the spread of the TB disease. To examine its impacts, an Epidemiological mathematical model with media efficacy parameter in the force of infection is established. Our study is an extension of the work [11]. In our research a mathematical model of TB transmission dynamics is constructed by considering the total population *N*(*t*) which is divided into six isolated compartments: Susceptible (*S*), Vaccinated population (*V*), Exposed or latent TB infected (*L*), Active TB infected (*I*), Populations undergoing for treatment (*T*) and Recovered population (*R*). The susceptible population increases by birth recruitment and vaccine waning rates *π*Λ and *ω*, respectively. These individuals are vaccinated with rate *q*, infected with mycobacterium tuberculosis with rate (1 − *c*)*η* and the remaining may progress to active TB episodes with infection rate *cη* where $\eta =(\beta_{1}-\frac{\beta_{2}I}{m+I})$, and *m* is media efficacy. The latent TB individuals progress into active TB infected class with rate *α*. The infected individual goes with treatment rate *γ* to the treatment class. The individuals in the undergoing for treatment class may successfully recover with effective treatment rate *pθ* or may come back into the infected class because of low level of treatment or treatment interruption with rate (1 − *p*)*θ*. The recovered individual comeback into susceptible class with rate *ρ* because of temporary immunity system. All individuals in each compartment decrease by natural death *μ*. There is a disease induced death rate *d* only in the infected compartment and wholly parameters are positive. In the force of infection [11], $\eta =(\beta_{1}-\frac{\beta_{2}I}{m+I})$ a continuous bounded term $\frac{I}{m+I}$ implies disease saturation or psychological effects reduction, *m* is the media efficacy where *m* > 0, *β*_1_ is transmission rate before media alert, *β*_2_ is transmission rate after media alert. To predict media influence, we looked at media efficacy (*m*), which measures the amount of information transmitted across all media types (such as print media, broadcast media, internet media, and outdoor media). Media effectiveness refers to the populations to which media information reaches. If *m* = 0, then the transmission rate is constant and the force of infection becomes *η* = *βI* as studied in many epidemiological TB models [16, 19, 34]. Accordingly, the flow chart of the model is displayed in Fig 1 and the deterministic mathematical model equations are determined in Eq (1).

Based on Fig 1 above the deterministic equations of the mathematical model has the form

$$\begin{array}{c} \begin{array}{cc} \frac{dS}{dt} & =\pi \Lambda -\eta SI-(\mu +q)S+\omega V+\rho R,\\ \frac{dV}{dt} & =(1-\pi )\Lambda +qS-(\omega +\mu )V,\\ \frac{dL}{dt} & =(1-c)\eta SI-(\mu +\alpha )L,\\ \frac{dI}{dt} & =c\eta SI+\alpha L+(1-p)\theta T-(\mu +\gamma +d)I,\\ \frac{dT}{dt} & =\gamma I-(\theta +\mu )T,\\ \frac{dR}{dt} & =p\theta T-(\mu +\rho )R, \end{array} \end{array}$$ (1)

with the initial condition *S*_0_ > 0, *V*_0_ > 0, *L*_0_ ≥ 0, *I*_0_ ≥ 0, *T*_0_ ≥ 0, *R*_0_ ≥ 0, and all parameters in the equation are positive. Tables 1 and 2 describes the state variables and the parameters of model (1), respectively.

**Table 1:** Description of the state variables.

State Variables	Descriptions
*S*	Individuals who are healthy, but they can be caught by the disease.
*V*	Individuals who are healthy, but they have taken TB vaccine.
*L*	Individuals who have TB bacteria, but they are not infectious & have no pain.
*I*	Individuals who have TB bacteria, can transmit the disease and have pain.
*T*	Individuals who are undergoing for treatment class.
*R*	Individuals who are recovered from TB and again who can susceptible.

**Table 2:** Description of the parameters.

Parameters	Descriptions
Λ	Recruitment rate by birth
*q*	Vaccination rate
*ω*	Vaccine waning rate
*μ*	Natural death rate
*d*	TB induced death rate for only the infected individual
*β*_1_	Transmission rate before media alert
*β*_2_	Transmission rate after media alert
*m*	Media efficacy
*α*	Rate of disease latency
*γ*	Detection rate
*θ*	Recovery rate with effective treatment
*ρ*	Re-infection rate or Weakening rate of immunity
*π*, *c*, *p*	Proportion

## 3 Analysis of the mathematical model

### 3.1 Wel-Posedness

The feasible region of the mathematical model is the region in which all populations are non-negative and bounded. For the model Eq (1) to be epidemiologically meaningful, it is necessary to ensure that all state variables of the model are non-negative for all time (*t*). To check the validity of the model (1), we need to prove the positivity and boundedness of the model.

#### 3.1.1 Positivity of the Solutions

**Theorem 3.1**. *Let the initial conditions S*(0) > 0, *V*(0) > 0, *L*(0) > 0, *I*(0) > 0, *T*(0) > 0, *R*(0) > 0; *then all the solutions* (*S*(*t*), *V*(*t*), *L*(*t*), *I*(*t*), *T*(*t*), *R*(*t*)) *of the model* (1) *are positive for all time t* > 0.

*Proof*. Let *τ* = sup{*t* > 0: *S*(*t*) > 0, *V*(*t*) > 0, *L*(*t*) > 0, *I*(*t*) > 0, *T*(*t*) > 0, *R*(*t*) > 0}. From the continuity of *S*(*t*), *V*(*t*), *L*(*t*), *I*(*t*), *T*(*t*), and *R*(*t*) we deduce that *τ* > 0 [35]. If *τ* = +∞, then positivity of the solutions are hold, but if 0 < *τ* < +∞, *S*(*t*) = 0 or *V*(*t*) = 0 or *L*(*t*) = 0 or *I*(*t*) = 0 or *T*(*t*) = 0 or *R*(*t*) = 0. Now from the above model Eq (1) we have the first equation is $\frac{dS}{dt}=\pi \Lambda -\eta SI-\left(\mu +q\right)S+\omega V+\rho R$ and it can be written as $\frac{dS}{dt}+\left(\eta I+\left(\mu +q\right)\right)S=\pi \Lambda +\omega V+\rho R$ which is a first-order linear ordinary differential equation. To solve this equation first find the integrating factor *IF* = *e*^∫(*ηI*+(*μ*+*q*))*dt*^, then multiplying the equation by the integrating factor *e*^∫(*ηI*+(*μ*+*q*))*dt*^, we will get the equation

$$e^{\int \left(\eta I+(\mu +q)\right)dt}(\frac{dS}{dt})+\left(\eta I+\left(\mu +q\right)\right)Se^{\int \left(\eta I+(\mu +q)\right)dt}=\left(\pi \Lambda +\omega V+\rho R\right)e^{\int \left(\eta I+(\mu +q)\right)dt}$$

$$\begin{array}{c} \begin{array}{cc} & \Rightarrow \frac{d(Se^{\int \left(\eta I+(\mu +q)\right)dt})}{dt}=(\pi \Lambda +\omega V+\rho R)e^{\int \eta Idt+(\mu +q)t},\\ & \Rightarrow S\left(\tau \right)e^{\int \left(\eta I+(\mu +q)\right)dt}=S\left(0\right)+\int_{0}^{\tau }\left(\pi \Lambda +\omega V+\rho R\right)e^{\int \left(\eta I+(\mu +q)\right)dt}dt,\\ & \Rightarrow S\left(\tau \right)=(S\left(0\right)+\int_{0}^{\tau }\left(\pi \Lambda +\omega V+\rho R\right)e^{\int \eta Idt+(\mu +q)t}dt)e^{-\int \left(\eta I+\mu +q\right)dt}>0, \end{array} \end{array}$$

for all *t* > 0. Hence *S*(*τ*)is positive for all *τ* > 0.

From the second equation;

$$\frac{dV}{dt}=\left(1-\pi \right)\Lambda +qS-\left(\omega +\mu \right)V$$

we obtain the form of linear first order ODE;

$$\frac{dV}{dt}+\left(\omega +\mu \right)V=\left(1-\pi \right)\Lambda +qS.$$

We note that, *e*^(*μ*+*ω*)*t*^ is the integrating factor and then after some calculation it implies that,

$$\begin{array}{c} \begin{array}{c} \Rightarrow V\left(\tau \right)=(V\left(0\right)+\int_{0}^{\tau }\left(\left(1-\pi \right)\Lambda +qS\right)e^{(\mu +\omega )t}dt)e^{-(\mu +\omega )t}>0. \end{array} \end{array}$$

Hence, *V*(*τ*)is positive for all *τ* > 0.

Similarly, the remaining equations of system (1) can be proved and gives us the following out puts:

$$\begin{array}{c} \begin{array}{cc} & \frac{dL}{dt}=\left(1-c\right)\eta SI-\left(\mu +\alpha \right)L,\\ & \Rightarrow L\left(\tau \right)=(L\left(0\right)+\int_{0}^{\tau }\left(1-c\right)\eta SIe^{(\mu +\alpha )t}dt)e^{-(\mu +\alpha )t}\;>\;0. \end{array} \end{array}$$

Hence, *L*(*τ*) is positive for all *τ* > 0.

$$\begin{array}{c} \begin{array}{cc} & \frac{dI}{dt}=c\eta SI+\alpha L+\left(1-p\right)\theta T-\left(\mu +\gamma +d\right)I,\\ \Rightarrow & I\left(\tau \right)=(I\left(0\right)+\int_{0}^{\tau }\left(\alpha L+\left(1-p\right)\theta T\right)e^{\int (\mu +\gamma +d)-c\eta Sdt}dt)e^{-\int (\mu +\gamma +d)-c\eta Sdt}\;>\;0. \end{array} \end{array}$$

Hence, *I*(*τ*) is positive for all *τ* > 0.

$$\begin{array}{c} \begin{array}{cc} & \frac{dT}{dt}=\gamma I-\left(\theta +\mu \right)T,\\ & \Rightarrow T\left(\tau \right)=(T\left(0\right)+\int_{0}^{\tau }\gamma e^{(\theta +\mu )t}Idt)e^{-(\theta +\mu )t}\;>\;0. \end{array} \end{array}$$

Hence, *T*(*τ*) is positive for all *τ* > 0.

$$\begin{array}{c} \begin{array}{cc} & \frac{dR}{dt}=p\theta T-\left(\mu +\rho \right)R,\\ & \Rightarrow R\left(\tau \right)=(R\left(0\right)+\int_{0}^{\tau }p\theta Te^{(\mu +\rho )t}dt)e^{-(\mu +\rho )t}\;>\;0,R\left(\tau \right)\;\text{is}\;\text{positive}\;\text{for}\;\text{all}\;\tau >0. \end{array} \end{array}$$

Hence, all solutions of the model (1) are positive for all *τ* ≥ 0.

#### 3.1.2 Boundedness of the model

**Theorem 3.2**. *The feasible region* Ω *of the mathematical model in* Eq (1) *is defined as*: $\Omega =\{\left(S\left(t\right),V\left(t\right),L\left(t\right),I\left(t\right),T\left(t\right),R\left(t\right)\right)\in R_{+}^{6}:N\left(t\right)\leq \frac{\Lambda }{\mu }\}$ *is positively invariant set of system* Eq (1).

*Proof*. The total population *N*(*t*) is given by

N(t)=V(t)+S(t)+L(t)+I(t)+T(t)+R(t),andhencethederivativeofN(t)becomes

$$\begin{array}{c} \begin{array}{cc} & \frac{dN(t)}{dt}=\;\frac{dV(t)}{dt}+\frac{dS(t)}{dt}+\frac{dL(t)}{dt}+\frac{dI(t)}{dt}+\frac{dT(t)}{dt}+\frac{dR(t)}{dt}. \end{array} \end{array}$$

This implies,

$$\begin{array}{c} \begin{array}{cc} & \frac{dN(t)}{dt}=\;\Lambda -\mu N\left(t\right)-dI \end{array} \end{array}$$

If there is no disease induced death rate then we obtain

$$\begin{array}{c} \begin{array}{cc} & \frac{dN(t)}{dt}\;\leq \;\Lambda -\mu N\left(t\right).\\ & \Rightarrow \frac{dN(t)}{dt}+\mu N\left(t\right)\;\leq \;\Lambda \\ & \Rightarrow N\left(t\right)\;\leq \;N_{0}e^{-\mu t}+\frac{\Lambda }{\mu }\left(1-e^{-\mu t}\right)\\ & \Rightarrow \;\text{As}\;t⟶\infty ,\;N\left(t\right)⟶\;\frac{\Lambda }{\mu }. \end{array} \end{array}$$

Hence, each solution of the system Eq (1) with initial condition, for all *t* ≥ 0 remains in

$$\begin{array}{c} \Omega =\{(S(t),V(t),L(t),I(t),T(t),R(t))\in R_{+}^{6}:N\left(t\right)\leq \frac{\Lambda }{\mu }\}. \end{array}$$ (2)

Therefore, the region Ω is positively invariant set and on this set the mathematical model is well posed epidemiologically and mathematically. Hence, it is sufficient to study the dynamics of the model in Ω.

### 3.2 Existence of disease free equilibrium point

#### 3.2.1 Disease Free Equilibrium Point (DFEP)

To get the disease free equilibrium point we need to make state variables is equal to zero in the mathematical model Eq (1). That is if *L* = *I* = 0 then *T* = *R* = 0. Hence, the remaining state variables value become $S=\frac{\Lambda }{\mu }(\frac{\pi \mu +\omega }{\mu +q+\omega })$ and $V=\frac{\Lambda }{\mu }(\frac{\left(q+(1-\pi )\mu \right)}{\left(q+\mu +\omega \right)})$. Therefore, the disease free equilibrium point is,

$$E^{0}\left(S^{0},V^{0},L^{0},I^{0},T^{0},R^{0}\right)=(\frac{\Lambda }{\mu }(\frac{\pi \mu +\omega }{\mu +q+\omega }),\frac{\Lambda }{\mu }(\frac{\left(q+(1-\pi )\mu \right)}{\left(q+\mu +\omega \right)}),0,0,0,0).$$

### 3.3 Effective reproduction number

Epidemiologically, the reproduction number of the disease tells us how many secondary cases one infected individual will produce in an entirely susceptible population during its period as an infective. In our model we will have both an effective (*R*_*eff*_) and basic reproduction number (*R*_0_) because of using vaccine as a prevention compartment. To get *R*_*eff*_ of the dynamical system (1), we proceed by considering the infected compartments only, as follows: Let *X* = (*L*(*t*), *I*(*t*), *T*(*t*)), *X* ≥ 0 be the vector of densities of individuals in each compartments and consider

$$\frac{dX}{dt}=F_{i}\left(X\right)-V_{i}\left(X\right),$$

where, *F*_*i*_(*X*) be the appearance of new infection in compartment i and *V*_*i*_(*X*) be the remaining transfer terms. Then, the effective reproduction number is evaluated as *R*_*eff*_ = *ρ*(*FV*^−1^), where *ρ*(*FV*^−1^) denotes the spectral radius of the generation matrix *FV*^−1^ (that is, the eigenvalue with the maximum absolute value). The dynamical system of the infected groups is,

$$\begin{array}{c} \begin{array}{cc} \frac{dL}{dt} & =\left(1-c\right)\lambda_{1}SI-\left(\mu +\alpha \right)L,\\ \frac{dI}{dt} & =c\lambda_{1}SI+\alpha L+\left(1-p\right)\theta T-\left(\mu +\gamma +d\right)I,\\ \frac{dT}{dt} & =\gamma I-(\theta +\mu )T. \end{array} \end{array}$$ (3)

Using the method in [36], then the transmission matrix *F* and the transition matrix *V* are given by: $f=(\begin{array}{c} (1-c)\eta SI\\ c\eta SI\\ 0 \end{array})\Rightarrow \frac{\partial f(X_{0})}{\partial x_{i}}=F=(\begin{array}{ccc} 0 & \left(1-c\right)\beta_{1}S^{0} & 0\\ 0 & c\beta_{1}S^{0} & 0\\ 0 & 0 & 0 \end{array}),$ and $v=(\begin{array}{c} (\mu +\alpha )L\\ (\mu +\gamma +d)I-\alpha L-(1-p)\theta T\\ (\theta +\mu )T-\gamma I \end{array})\Rightarrow \frac{\partial v(X_{0})}{\partial x_{i}}=V=(\begin{array}{ccc} \mu +\alpha & 0 & 0\\ -\alpha & \mu +\gamma +d & -(1-p)\theta \\ 0 & -\gamma & \mu +\theta \end{array})$ In this work where, *x*_*i*_ = {*L*, *I*, *T*} and *X*_0_ = {*S*^0^, *V*^0^, *L*^0^, *I*^0^, *T*^0^, *R*^0^}. After substitution these points we obtain $F=(\begin{array}{ccc} 0 & \left(1-c\right)\beta_{1}\frac{\Lambda }{\mu }(\frac{\mu \pi +\omega }{\mu +q+\omega }) & 0\\ 0 & c\beta_{1}\frac{\Lambda }{\mu }(\frac{\mu \pi }{\mu +q+\omega }) & 0\\ 0 & 0 & 0 \end{array})$ and $V=(\begin{array}{ccc} \mu +\alpha & 0 & 0\\ -\alpha & \mu +\gamma +d & -(1-p)\theta \\ 0 & -\gamma & \mu +\theta \end{array})$.

To find the basic reproduction number (*R*_0_) and effective reproduction number (*R*_*eff*_) we need to find the matrix *V*^−1^. Since det(*V*) = |*V*| = (*μ* + *α*)((*μ* + *γ* + *d*)(*μ* + *θ*) − (1−*p*)*θγ*) ≠ 0, *V*^−1^ exists and is obtained as

$$V^{-1}=(\begin{array}{ccc} \frac{1}{\mu +\alpha } & 0 & 0\\ \frac{\alpha (\mu +\theta )}{\left(\mu +\alpha )((\mu +\gamma +d)(\mu +\theta )-(1-p)\theta \gamma \right)} & \frac{\mu +\theta }{\left((\mu +\gamma +d)(\mu +\theta )-(1-p)\theta \gamma \right)} & \frac{(1-p)\theta }{\left((\mu +\gamma +d)(\mu +\theta )-(1-p)\theta \gamma \right)}\\ \frac{\alpha \gamma }{\left(\mu +\alpha )((\mu +\gamma +d)(\mu +\theta )-(1-p)\theta \gamma \right)} & \frac{\gamma }{\left((\mu +\gamma +d)(\mu +\theta )-(1-p)\theta \gamma \right)} & \frac{\mu +\gamma +d}{\left((\mu +\gamma +d)(\mu +\theta )-(1-p)\theta \gamma \right)} \end{array}).$$

Then, the required generation matrix is given by:

$$FV^{-1}=(\begin{array}{ccc} \frac{\left(1-c\right)\alpha \left(\mu +\theta \right)\beta_{1}S^{0}}{\left(\mu +\alpha )((\mu +\gamma +d)(\mu +\theta )-(1-p)\theta \gamma \right)} & \frac{\left(1-c\right)\left(\mu +\theta \right)\beta_{1}S^{0}}{\left((\mu +\gamma +d)(\mu +\theta )-(1-p)\theta \gamma \right)} & \frac{\left(1-c\right)\left(1-p\right)\theta \beta_{1}S^{0}}{\left((\mu +\gamma +d)(\mu +\theta )-(1-p)\theta \gamma \right)}\\ \frac{c\alpha \left(\mu +\theta \right)\beta_{1}S^{0}}{\left(\mu +\alpha )((\mu +\gamma +d)(\mu +\theta )-(1-p)\theta \gamma \right)} & \frac{c\left(\mu +\theta \right)\beta_{1}S^{0}}{\left((\mu +\gamma +d)(\mu +\theta )-(1-p)\theta \gamma \right)} & \frac{c\left(1-p\right)\theta \beta_{1}S^{0}}{\left((\mu +\gamma +d)(\mu +\theta )-(1-p)\theta \gamma \right)}\\ 0 & 0 & 0 \end{array}).$$

Now we can calculate the reproduction number by using the formula; *R*_0_ = *ρ*(*FV*^−1^) which is the largest eigenvalue of *FV*^−1^ in magnitude. Therefor, after simplification our effective reproduction number (*R*_*eff*_) is

$$R_{eff}=\frac{\beta_{1}\Lambda \left(\mu +\theta \right)\left(\mu c+\alpha \right)\left(\mu \pi +\omega \right)}{\mu \left(\mu +\alpha \right)\left(\mu +q+\omega \right)((\mu +\theta )(\mu +\gamma +d)-(1-p)\theta \gamma )}.$$

The basic reproduction number (*R*_0_) also calculated with out vaccination by setting *q* = *ω* = 0 and it becomes

$$R_{0}=\frac{\beta_{1}\Lambda \left(\mu +\theta \right)\left(\mu c+\alpha \right)}{\mu \left(\mu +\alpha \right)((\mu +\theta )(\mu +\gamma +d)-(1-p)\theta \gamma )}.$$

This quantity determines whether the TB infection will increase, die out, or remain constant. If *R*_*eff*_ > 1, then each infected individual produces, on average, more than one new infection, and the disease can invade the population. If *R*_*eff*_ < 1, then on average, an infected individual produces less than one new infected individual over the course of its infectious period, however this may not sufficient to eradicate TB from the community because the bifurcation may exist and it needs more analysis. If *R*_*eff*_ = 1, then each infected individual will infect on average exactly one other new infected individual. Thus, the effective reproduction number *R*_*eff*_ is often considered as the threshold quantity that determines when an infection can invade and persist in a new host population.

### 3.4 Local stability analysis of disease free equilbrium point (DFEP)

The Jacobian matrix of the mathematical model Eq (1) is evaluated as;

$$J\left(S,V,L,I,T,R\right)=(\begin{array}{cccccc} \frac{\partial f_{1}}{\partial S} & \frac{\partial f_{1}}{\partial V} & \frac{\partial f_{1}}{\partial L} & \frac{\partial f_{1}}{\partial I} & \frac{\partial f_{1}}{\partial T} & \frac{\partial f_{1}}{\partial R}\\ \frac{\partial f_{2}}{\partial S} & \frac{\partial f_{2}}{\partial V} & \frac{\partial f_{2}}{\partial L} & \frac{\partial f_{2}}{\partial I} & \frac{\partial f_{2}}{\partial T} & \frac{\partial f_{2}}{\partial R}\\ \frac{\partial f_{3}}{\partial S} & \frac{\partial f_{3}}{\partial V} & \frac{\partial f_{3}}{\partial L} & \frac{\partial f_{3}}{\partial I} & \frac{\partial f_{3}}{\partial T} & \frac{\partial f_{3}}{\partial R}\\ \frac{\partial f_{4}}{\partial S} & \frac{\partial f_{4}}{\partial V} & \frac{\partial f_{4}}{\partial L} & \frac{\partial f_{4}}{\partial I} & \frac{\partial f_{4}}{\partial T} & \frac{\partial f_{4}}{\partial R}\\ \frac{\partial f_{5}}{\partial S} & \frac{\partial f_{5}}{\partial V} & \frac{\partial f_{5}}{\partial L} & \frac{\partial f_{5}}{\partial I} & \frac{\partial f_{5}}{\partial T} & \frac{\partial f_{5}}{\partial R}\\ \frac{\partial f_{6}}{\partial S} & \frac{\partial f_{6}}{\partial V} & \frac{\partial f_{6}}{\partial L} & \frac{\partial f_{6}}{\partial I} & \frac{\partial f_{6}}{\partial T} & \frac{\partial f_{6}}{\partial R} \end{array})$$

Then, the Jacobian matrix of system Eq (1) at state variables, *J*(*X*) = *J*(*S*, *V*, *L*, *I*, *T*, *R*) becomes,

$$\begin{array}{c} J\left(X\right)=(\begin{array}{cccccc} -\eta I-(\mu +q) & \omega & 0 & -\phi S & 0 & \rho \\ q & -(\mu +\omega ) & 0 & 0 & 0 & 0\\ (1-c)\eta I & 0 & -(\mu +\alpha ) & (1-c)\phi S & 0 & 0\\ c\eta I & 0 & \alpha & c\phi S-(\mu +\gamma +d) & (1-p)\theta & 0\\ 0 & 0 & 0 & \gamma & -(\mu +\theta ) & 0\\ 0 & 0 & 0 & 0 & p\theta & -(\mu +\rho ) \end{array}) \end{array}$$ (4)

where, $\phi =(\frac{m\left(\beta_{1}m+2\left(\beta_{1}-\beta_{2}\right)I\right)+\left(\beta_{1}-\beta_{2}\right)I^{2}}{{\left(m+I\right)}^{2}})$.

**Theorem 3.3**. *The DFE is locally asymptotically stable if R*_*eff*_ < 1 *and unstable otherwise*.

*Proof*. The local stability of the DFE equilibrium is determined by the sign of eigenvalues of the Jacobean matrix computed at this point. The Jacobian matrix at *E*^0^, where

$$E^{0}=\left(S^{0},V^{0},L^{0},I^{0},T^{0},R^{0}\right)=(\frac{\Lambda }{\mu }(\frac{\pi \mu +\omega }{\mu +q+\omega }),\frac{\Lambda }{\mu }(\frac{\left(q+(1-\pi )\mu \right)}{\left(q+\mu +\omega \right)}),0,0,0,0)\;\text{is}$$

$$J\left(E^{0}\right)=(\begin{array}{cccccc} -(\mu +q) & \omega & 0 & -\beta_{1}S^{0} & 0 & \rho \\ q & -(\mu +\omega ) & 0 & 0 & 0 & 0\\ 0 & 0 & -(\mu +\alpha ) & \left(1-c\right)\beta_{1}S^{0} & 0 & 0\\ 0 & 0 & \alpha & c\beta_{1}S^{0}-\left(\mu +\gamma +d\right) & (1-p)\theta & 0\\ 0 & 0 & 0 & \gamma & -(\mu +\theta ) & 0\\ 0 & 0 & 0 & 0 & p\theta & -(\mu +\rho ) \end{array}).$$

Then, the eigen values of *J*(*E*^0^) are obtine from the equation det(*J*(*E*^0^) − λ*I*) = 0. That is,

$$|\begin{array}{cccccc} -(\mu +q)-\lambda & \omega & 0 & -(\beta_{1}S^{0}) & 0 & \rho \\ q & -(\mu +\omega )-\lambda & 0 & 0 & 0 & 0\\ 0 & 0 & -(\mu +\alpha )-\lambda & \left(1-c\right)\beta_{1}S^{0} & 0 & 0\\ 0 & 0 & \alpha & c\beta_{1}S^{0}-\left(\mu +\gamma +d\right)-\lambda & (1-p)\theta & 0\\ 0 & 0 & 0 & \gamma & -(\mu +\theta )-\lambda & 0\\ 0 & 0 & 0 & 0 & p\theta & -(\mu +\rho )-\lambda \end{array}|=0.$$

This implies

(-(μ+q)-λ)(-(μ+ω)-λ)-ωq=0(whichisquadraticequation),

or

$$\begin{array}{c} |\begin{array}{cccc} -(\mu +\alpha )-\lambda & \left(1-c\right)\beta_{1}S^{0} & 0 & 0\\ \alpha & c\beta_{1}S^{0}-\left(\mu +\gamma +d\right)-\lambda & (1-p)\theta & 0\\ 0 & \gamma & -(\mu +\theta )-\lambda & 0\\ 0 & 0 & p\theta & -(\mu +\rho )-\lambda \end{array}|=0. \end{array}$$

The quadratic equation

$$\left(-\left(\mu +q\right)-\lambda \right)\left(-\left(\mu +\omega \right)-\lambda \right)-\omega q=0\Rightarrow \lambda^{2}+((\mu +q)+(\mu +\omega ))\lambda +\mu \left(\mu +q+\omega \right)\;=\;0.$$

From this we have two roots or eigenvalues, i.e., $\lambda_{1}=\frac{-((\mu +\omega )+(\mu +q))-\sqrt{{\left((\mu +\omega )+(\mu +q)\right)}^{2}-4\mu \left(\mu +q+\omega \right)}}{2}=-\left(\mu +q+\omega \right)<0$, and $\lambda_{2}=\frac{-((\mu +\omega )+(\mu +q))+\sqrt{{\left((\mu +\omega )+(\mu +q)\right)}^{2}-4\mu \left(\mu +q+\omega \right)}}{2}=-\mu <0$.

And, the remaining eigenvalues are calculated as follows:

$$\begin{array}{c} |\begin{array}{cccc} -(\mu +\alpha )-\lambda & \left(1-c\right)\beta_{1}S^{0} & 0 & 0\\ \alpha & c\beta_{1}S^{0}-\left(\mu +\gamma +d\right)-\lambda & (1-p)\theta & 0\\ 0 & \gamma & -(\mu +\theta )-\lambda & 0\\ 0 & 0 & p\theta & -(\mu +\rho )-\lambda \end{array}|=0. \end{array}$$

⇒((*μ* + *ρ*) + λ) = 0 or (((*μ* + *α*) + λ)(λ + (*μ* + *γ* + *d*) − *cβ*_1_*S*^0^) − (1 − *c*)*β*_1_*S*^0^)((*μ* + *θ*) + λ) − ((*μ* + *α*) + λ)((1 − *p*)*θ*)(*γ*) = 0.

After some simplifications we get λ_3_ = −(*μ* + *ρ*) < 0 and the remaining eigen values are obtained in the equation

$$\begin{array}{c} \lambda^{3}+a_{2}\lambda^{2}+a_{1}\lambda +a_{0}=0. \end{array}$$ (5)

The sign of other eigenvalues λ_4_, λ_5_, λ_6_ are identified from Eq (5) using the Routh -Hurwith criteria technique. We also note that, $a_{2}=\left(\mu +\alpha \right)+\left(\mu +\theta \right)+\left(\mu +\gamma +d\right)-c\beta_{1}\frac{\Lambda }{\mu }(\frac{\pi \mu +\omega }{\mu +q+\omega })>0$ if *μ*(*μ* + *q* + *ω*)((*μ* + *α*) + (*μ* + *θ*) + (*μ* + *γ* + *d*)) > *cβ*_1_Λ(*πμ* + *ω*), $a_{1}=\left(\mu +\theta \right)\left(\mu +\alpha \right)-((\mu +\alpha )+(\mu +\theta ))(c\beta_{1}\frac{\Lambda }{\mu }(\frac{\pi \mu +\omega }{\mu +q+\omega })-\left(\mu +\gamma +d\right))-\alpha \left(1-c\right)\beta_{1}\frac{\Lambda }{\mu }(\frac{\pi \mu +\omega }{\mu +q+\omega })-\left(1-p\right)\theta \gamma$, and $a_{0}=-\left(\mu +\theta \right)\left(\mu +\alpha \right)(c\beta_{1}\frac{\Lambda }{\mu }(\frac{\pi \mu +\omega }{\mu +q+\omega })-\left(\mu +\gamma +d\right))-\left(1-p\right)\theta \gamma \left(\mu +\alpha \right)-\left(\mu +\theta \right)\alpha \left(1-c\right)\beta_{1}\frac{\Lambda }{\mu }(\frac{\pi \mu +\omega }{\mu +q+\omega })$.

We can also rewrite *a*_0_ in terms of the effective reproduction number (*R*_*eff*_) as, $a_{0}=\frac{\left(\mu +\theta \right)\left(\mu c+\alpha \right)\beta_{1}S^{0}\left(1-R_{eff}\right)}{R_{eff}}=\frac{\left(\mu +\theta \right)\left(\mu c+\alpha \right)\left(\mu \pi +\omega \right)\beta_{1}\Lambda \left(1-R_{eff}\right)}{\mu \left(\mu +q+\omega \right)R_{eff}}>0,\;\text{if}\;R_{eff}<1.$ From the above we get the eigenvalues as the form: λ_1_ = −(*μ* + *q* + *ω*) < 0, λ_2_ = −*μ* < 0, λ_3_ = −(*μ* + *ρ*) < 0, and the remaining eigenvalues (λ_4_, λ_5_ and λ_6_) are the roots of Eq (5). Now, to determine the sign of the roots of Eq (5) we use the Routh-Hurwitz stability criterion [36].

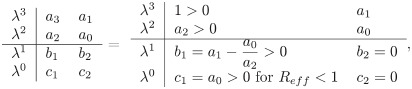

where $b_{1}=\frac{-1}{a_{2}}|\begin{array}{cc} a_{3} & a_{1}\\ a_{2} & a_{0} \end{array}|$ $b_{1}=\frac{-1}{a_{2}}(a_{0}a_{3}-a_{2}a_{1})=a_{1}-\frac{a_{0}}{a_{2}}>0$ for $a_{1}>\frac{a_{0}}{a_{2}}$ and *b*_2_ = 0, $c_{1}=\frac{-1}{b_{1}}|\begin{array}{cc} a_{2} & a_{0}\\ b_{1} & b_{2} \end{array}|=a_{0}$.

Then, the above Routh-Hurwitz array shows that in the first column of the array, there is no sign change, which means all the first column elements are positive for *R*_*eff*_ < 1. In the Routh-Hurwitz stability criterion, the necessary condition is that all roots have a negative real part and that all elements of the first column of the array have the same sign. Therefore, the necessary condition is satisfied, and hence the sign of the remaining eigenvalues (λ_4_, λ_5_, & λ_6_) is negative for *R*_*eff*_ < 1. Hence, the disease-free equilibrium point is locally asymptotically stable for *R*_*eff*_ < 1 and unstable otherwise.

### 3.5 Local stability analysis of Endemic Equilibrium Point (EEP)

#### 3.5.1 Existence of EEP

An endemic equilibrium point E*(S*, V*, L*, I*, T*, R*) is a point where the derivatives vanish, that is,

$$\begin{array}{c} \begin{array}{cc} \frac{dS}{dt} & =\pi \Lambda -\eta SI-(\mu +q)S+\omega V+\rho R=0,\\ \frac{dV}{dt} & =(1-\pi )\Lambda +qS-(\omega +\mu )V=0,\\ \frac{dL}{dt} & =(1-c)\eta SI-(\mu +\alpha )L=0,\\ \frac{dI}{dt} & =c\eta SI+\alpha L+(1-p)\theta T-(\mu +\gamma +d)I=0,\\ \frac{dT}{dt} & =\gamma I-(\theta +\mu )T=0,\\ \frac{dR}{dt} & =p\theta T-(\mu +\rho )R=0. \end{array} \end{array}$$ (6)

From the second equation of system (6),

$$\begin{array}{c} \begin{array}{ccc} & \frac{dV}{dt}=\left(1-\pi \right)\Lambda +qS-\left(\omega +\mu \right)V=0, & \Rightarrow V=\frac{(1-\pi )\Lambda +qS}{\left(\omega +\mu \right)}. \end{array} \end{array}$$

From the third equation of system (6),

$$\begin{array}{c} \begin{array}{ccc} & \frac{dL}{dt}=\left(1-c\right)\eta SI-\left(\mu +\alpha \right)L=0,\;\;\; & \Rightarrow L=\frac{(1-c)\eta SI}{\left(\mu +\alpha \right)}. \end{array} \end{array}$$

From fifth equation of system (6),

$$\begin{array}{c} \begin{array}{ccc} & \frac{dT}{dt}=\gamma I-\left(\theta +\mu \right)T=0,\;\;\;\;\;\;\;\;\;\;\;\;\;\;\; & \Rightarrow T=\frac{\gamma I}{\left(\theta +\mu \right)}.\;\;\; \end{array} \end{array}$$

From sixth equation of system (6),

$$\begin{array}{c} \begin{array}{ccc} & \frac{dR}{dt}=p\theta T-\left(\mu +\rho \right)R=0,\;\;\;\;\;\;\;\;\; & \Rightarrow R=\frac{\theta \gamma I}{\left(\mu +\rho )(\theta +\mu \right)}. \end{array} \end{array}$$

The fourth equation of system (6)

$$\frac{dI}{dt}=c\eta SI+\alpha L+\left(1-p\right)\theta T-\left(\mu +\gamma +d\right)I=0,$$

with $T=\frac{\gamma I}{\left(\theta +\mu \right)}$ and $L=\frac{(1-c)\eta SI}{\left(\mu +\alpha \right)}$ becomes;

$$\begin{array}{c} \begin{array}{cc} \Rightarrow & c\eta SI+\alpha L+(1-p)\theta T-(\mu +\gamma +d)I=0\\ \Rightarrow & c\eta SI+\alpha \frac{(1-c)\eta SI}{\left(\mu +\alpha \right)}+\left(1-p\right)\theta \frac{\gamma I}{\left(\theta +\mu \right)}-\left(\mu +\gamma +d\right)I=0\\ \Rightarrow & S=\frac{k}{\lambda_{1}},\;\text{where}\;k=\frac{\left(\mu +\alpha \right)((\theta +\mu )(\mu +\gamma +d)-(1-p)\theta \gamma )}{\left(\mu c+\alpha )(\theta +\mu \right)}. \end{array} \end{array}$$

The first equation of system (6)

$$\frac{dS}{dt}=\pi \Lambda -\eta SI-\left(\mu +q\right)S+\omega V+\rho R=0,$$

with $S=\frac{k}{\eta },\;\;V=\frac{(1-\pi )\Lambda +qS}{\left(\omega +\mu \right)}$ and $R=\frac{\theta \gamma I}{\left(\mu +\rho )(\theta +\mu \right)}$ becomes;

$$\begin{array}{c} \begin{array}{cc} \Rightarrow & \pi \Lambda -\eta SI-(\mu +q)S+\omega V+\rho R=0\\ \Rightarrow & (\frac{(\pi (\omega +\mu )+\omega (1-\pi ))\Lambda \eta +\omega qk}{(\omega +\mu )\eta })-\frac{(\mu +q)k}{\eta }-(\frac{k(\mu +\rho )(\theta +\mu )-\rho \theta \gamma }{\left(\mu +\rho )(\theta +\mu \right)})I=0\\ \Rightarrow & \left(\frac{k(\mu +\rho )(\theta +\mu )-\rho \theta \gamma }{\left(\mu +\rho )(\theta +\mu \right)})I=(\frac{(\mu \pi +\omega )\Lambda \eta -\mu \left(\mu +q+\omega \right)k}{(\omega +\mu )\eta }\right) \end{array} \end{array}$$

Let $A=\frac{(\mu +\theta )(\mu +\rho )(\mu \pi +\omega )\Lambda }{\left(\mu +\omega \right)(k(\mu +\theta )(\mu +\rho )-\rho \gamma p\theta )}$, $B=\frac{\left(\mu +\theta )(\mu +\rho )(q+\mu +\omega )(\mu k\right)}{\left(\mu +\omega )(k(\mu +\theta )(\mu +\rho )-\rho \gamma p\theta \right)}$, and $\eta^{*}=\beta_{1}-\beta_{2}\frac{I^{*}}{m+I^{*}}$.

$$\begin{array}{c} \begin{array}{cc} \Rightarrow & I=A-\frac{B}{\eta }=A-\frac{B}{\beta_{1}-\beta_{2}\frac{I}{m+I}}\\ \Rightarrow & \left(\beta_{1}-\beta_{2}\right)I^{2}+(B+\beta_{1}m+B-\left(\beta_{1}-\beta_{2}\right)A)I+\left(B-\beta_{1}A\right)m=0. \end{array} \end{array}$$

Then we get second degree quadratic equation as a function of I*, i.e.,

$$a_{2}{I^{*}}^{2}+a_{1}I^{*}+a_{0}=0,$$

where *a*_2_ = (*β*_1_ − *β*_2_), *a*_1_ = (*β*_1_*m* + *B* − (*β*_1_ − *β*_2_)*A*), and *a*_0_ = (*B* − *β*_1_*A*)*m*. Then the endemic equilibrium point is E*(S*, V*, L*, I*, T*, R*) where, $V^{*}=\frac{\left(1-\pi \right)\Lambda \eta^{*}+qk}{\left(mu+\omega \right)\eta^{*}}$, $S^{*}=\frac{k}{\eta^{*}}$, $L^{*}=\frac{(1-c)k}{\left(\alpha +\mu \right)}I^{*}$, $I^{*}=A-\frac{B}{\eta^{*}}$, $T^{*}=\frac{\gamma }{\mu +\theta }I^{*}$, $R^{*}=\frac{\gamma p\theta }{\left(\mu +\theta )(\mu +\rho \right)}I^{*}$, and the constant $k=\frac{\left(\alpha +\mu \right)[(\mu +\theta )(\mu +d+\gamma )-(1-p)\theta \gamma ]}{\left(\mu +\theta )(\mu c+\alpha \right)}$, $A=\frac{(\mu +\theta )(\mu +\rho )(\mu \pi +\omega )\Lambda }{\left(\mu +\omega \right)(k(\mu +\theta )(\mu +\rho )-\rho \gamma p\theta )}$, $B=\frac{\left(\mu +\theta )(\mu +\rho )(q+\mu +\omega )(\mu k\right)}{\left(\mu +\omega )(k(\mu +\theta )(\mu +\rho )-\rho \gamma p\theta \right)}$, $\lambda_{1}^{*}=\beta_{1}-\beta_{2}\frac{I^{*}}{m+I^{*}}$, and I* is a positive root(s) of the quadratic equation

$$\begin{array}{c} a_{2}{I^{*}}^{2}+a_{1}I^{*}+a_{0}=0. \end{array}$$ (7)

The leading coefficient of the above quadratic equation *a*_2_ = (*β*_1_ − *β*_2_) > 0, because the transmission contact rate before media alert (*β*_1_) is greater than the transmission contact rate after media alert (*β*_2_). Also,

$$a_{0}=\left(B-A\beta_{1}\right)m=(\frac{\beta_{1}\Lambda \left(\mu \pi +\omega \right)\left(\mu +\rho \right)\left(\mu +\theta \right)\left(1-R_{eff}\right)}{R_{eff}\left(\mu +\omega \right)\left[k\left(\mu +\rho \right)\left(\mu +\theta \right)-\rho p\theta \gamma \right]})m>0,\;\text{for}\;R_{eff}<1.$$

To determine the sign of the roots of *a*_2_I*^2^ + *a*_1_I* + *a*_0_ = 0, we use Descartes’ rule of signs [37, 38].

**Theorem 3.4**. *The dynamical system of the mathematical model* (1) *has the following conditions*:

1. *The mathematical model* (1) *has unique positive endemic equilibrium point if R*_*eff*_ > 1 *and for the conditions*;
  *(a) a*_1_ < 0 *and*
  *(b) a*_1_ > 0
2. *The mathematical model* (1) *has two positive endemic equilibrium points when R*_*eff*_ < 1 *and a*_1_ < 0.

Here we observe (Theorem 3.4) condition (2) shows that a bifurcation exists. That means the locally asymptotically stable disease-free equilibrium point co-exists with a locally asymptotically stable endemic equilibrium point if *R*_*eff*_ < 1. Epidemiologically existence of bifurcation shows the reproductive number less than one is not enough to eradicate the disease [39]. This indicates *R*_*eff*_ < 1 is the necessary condition but not sufficient condition to eradicate TB from the population. Center manifold theory has been used to decide the local stability of a non-hyperbolic equilibrium point. Carlos Castillo-Chavez and his collaborator Song have explored bifurcation in their mathematical modeling work which is based on the general center manifold theory. The method ensures easy implementation and guarantees the necessary and sufficient condition for backward bifurcation. This leads us to the bifurcation analysis of the model (1) by using Castillo-Chavez and Song theorem [39].

#### 3.5.2 Bifurcation analysis at EEP

Using the same procedure to the study [35], let us reset the state variables of model (1) (*S*, *V*, *L*, *I*, *T*, *R*) by the new variables (*x*_1_, *x*_2_, *x*_3_, *x*_4_, *x*_5_, *x*_6_). We can express these state variables as a vector form by *X* = (*x*_1_, *x*_2_, *x*_3_, *x*_4_, *x*_5_, *x*_6_)^*T*^ and let *F* = (*f*_1_, *f*_2_, *f*_3_, *f*_4_, *f*_5_, *f*_6_)^*T*^. Then the above mathematical model system Eq (1) can be rewritten as the form $\frac{dX}{dt}=F\left(X\right)$. Now the ODEs becomes;

$$\begin{array}{c} \begin{array}{cc} \frac{dx_{1}}{dt} & =\pi \Lambda -\eta x_{1}x_{4}-\left(\mu +q\right)x_{1}+\omega x_{2}+\rho x_{6}=f_{1},\\ \frac{dx_{2}}{dt} & =\left(1-\pi \right)\Lambda +qx_{1}-\left(\omega +\mu \right)x_{2}=f_{2},\\ \frac{dx_{3}}{dt} & =\left(1-c\right)\eta x_{1}x_{4}-\left(\mu +\alpha \right)x_{3}=f_{3},\\ \frac{dx_{4}}{dt} & =c\eta x_{1}x_{4}+\alpha x_{3}+\left(1-p\right)\theta x_{5}-\left(\mu +\gamma +d\right)x_{4}=f_{4},\\ \frac{dx_{5}}{dt} & =\gamma x_{4}-\left(\theta +\mu \right)x_{5}=f_{5},\\ \frac{dx_{6}}{dt} & =p\theta x_{5}-\left(\mu +\rho \right)x_{6}=f_{6}, \end{array} \end{array}$$ (8)

where $\eta =(\beta_{1}-\beta_{2}\frac{x_{4}}{m+x_{4}})$. The Jacobian matrix at DFE point *E*^0^, is defined as

$$J\left(E^{0}\right)=(\begin{array}{cccccc} -(\mu +q) & \omega & 0 & -\beta_{1}S^{0} & 0 & \rho \\ q & -(\mu +\omega ) & 0 & 0 & 0 & 0\\ 0 & 0 & -(\mu +\alpha ) & \left(1-c\right)\beta_{1}S^{0} & 0 & 0\\ 0 & 0 & \alpha & c\beta_{1}S^{0}-\left(\mu +\gamma +d\right) & (1-p)\theta & 0\\ 0 & 0 & 0 & \gamma & -(\mu +\theta ) & 0\\ 0 & 0 & 0 & 0 & p\theta & -(\mu +\rho ) \end{array}).$$

Now let us consider, *R*_0_ = 1 and suppose that $\beta =\beta_{1}^{*}$ is chosen as a bifurcation parameter. From *R*_0_ = 1 we can solve $\beta_{1}^{*}$;

$$\begin{array}{c} \begin{array}{cc} & R_{eff}=\frac{\beta_{1}\Lambda \left(\mu +\theta \right)\left(\mu c+\alpha \right)\left(\mu \pi +\omega \right)}{\mu \left(\mu +\alpha \right)\left(\mu +q+\omega \right)((\mu +\theta )(\mu +\gamma +d)-(1-p)\theta \gamma )}=1\\ \Rightarrow & \beta_{1}^{*}\Lambda \left(\mu +\theta \right)\left(\mu c+\alpha \right)\left(\mu \pi +\omega \right)=\mu \left(\mu +\alpha \right)\left(\mu +q+\omega \right)((\mu +\theta )(\mu +\gamma +d)-(1-p)\theta \gamma )\\ \Rightarrow & \beta_{1}^{*}=\frac{\mu \left(\mu +\alpha \right)\left(\mu +q+\omega \right)((\mu +\theta )(\mu +\gamma +d)-(1-p)\theta \gamma )}{\Lambda (\mu +\theta )(\mu c+\alpha )(\mu \pi +\omega )}. \end{array} \end{array}$$

Then the Jacobian matrix at the bifurcation parameter $\beta =\beta_{1}^{*}$ is defined as;

$$J\left(E^{0},\beta_{1}^{*}\right)=(\begin{array}{cccccc} -(\mu +q) & \omega & 0 & -\beta_{1}^{*}S^{0} & 0 & \rho \\ q & -(\mu +\omega ) & 0 & 0 & 0 & 0\\ 0 & 0 & -(\mu +\alpha ) & \left(1-c\right)\beta_{1}^{*}S^{0} & 0 & 0\\ 0 & 0 & \alpha & c\beta_{1^{*}}S^{0}-\left(\mu +\gamma +d\right) & (1-p)\theta & 0\\ 0 & 0 & 0 & \gamma & -(\mu +\theta ) & 0\\ 0 & 0 & 0 & 0 & p\theta & -(\mu +\rho ) \end{array}).$$

The eigenvalues of $J(\beta_{1}^{*})$ is calculated from the equation; $|J(E^{0},\beta_{1}^{*})-\lambda I|=0$ which implies,

$$|\begin{array}{cccccc} -(\mu +q) & \omega & 0 & -\beta_{1}^{*}S^{0} & 0 & \rho \\ q & -(\mu +\omega ) & 0 & 0 & 0 & 0\\ 0 & 0 & -(\mu +\alpha ) & \left(1-c\right)\beta_{1}^{*}S^{0} & 0 & 0\\ 0 & 0 & \alpha & c\beta_{1}^{*}S^{0}-\left(\mu +\gamma +d\right) & (1-p)\theta & 0\\ 0 & 0 & 0 & \gamma & -(\mu +\theta ) & 0\\ 0 & 0 & 0 & 0 & p\theta & -(\mu +\rho ) \end{array}|=0$$

After some simplification (it has similar calculation with in the previous section) we will have

⇒[(-(μ+ω)-λ)(-(μ+q)-λ)-ωq]((μ+ρ)+λ)=0,

$$\;\text{or}\;\;\;\;\;\;\;\;\;\;\;a_{3}\lambda^{3}+a_{2}\lambda^{2}+a_{1}\lambda +a_{0}=0$$

where, $a_{3}=1,a_{2}=\left(\mu +\alpha \right)+\left(\mu +\theta \right)+\left(\mu +\gamma +d\right)-c\beta_{1}^{*}S^{0}$, $a_{1}=-((\mu +\alpha )+(\mu +\theta ))(c\beta_{1}^{*}S^{0}-\left(\mu +\gamma +d\right))-\alpha \left(1-c\right)\beta_{1}^{*}S^{0}-\left(1-p\right)\theta \gamma +\left(\mu +\theta \right)\left(\mu +\alpha \right)$, and $a_{0}=-\left(\mu +\theta \right)\left(\mu +\alpha \right)(c\beta_{1}^{*}\frac{\Lambda }{\mu }(\frac{\pi \mu +\omega }{\mu +q+\omega })-\left(\mu +\gamma +d\right))-\left(1-p\right)\theta \gamma \left(\mu +\alpha \right)-\left(\mu +\theta \right)\alpha \left(1-c\right)\beta_{1}^{*}\frac{\Lambda }{\mu }(\frac{\pi \mu +\omega }{\mu +q+\omega })$, for $\beta_{1}^{*}=\frac{\mu \left(\mu +\alpha \right)\left(\mu +q+\omega \right)((\mu +\theta )(\mu +\gamma +d)-(1-p)\theta \gamma )}{\Lambda (\mu +\theta )(\mu c+\alpha )(\mu \pi +\omega )},a_{0}=0$.

So the above equation has one zero eigenvalue and it becomes

λ=0or

$\lambda^{2}+(\left(\mu +\alpha \right)+\left(\mu +\theta \right)+\left(\mu +\gamma +d\right)-c\beta_{1}^{*}S^{0})\lambda -((\mu +\alpha )+(\mu +\theta ))(c\beta_{1}^{*}S^{0}-\left(\mu +\gamma +d\right))-\alpha \left(1-c\right)\beta_{1}^{*}S^{0}-\left(1-p\right)\theta \gamma +\left(\mu +\theta \right)\left(\mu +\alpha \right)=0$.

$$\Rightarrow a_{2}\lambda^{2}+a_{1}\lambda +a_{0}=0,$$

where $a_{2}=1,a_{1}=\left(\mu +\alpha \right)+\left(\mu +\theta \right)+\left(\mu +\gamma +d\right)-c\beta_{1}^{*}S^{0}$, $a_{0}=-((\mu +\alpha )+(\mu +\theta ))(c\beta_{1}^{*}S^{0}-\left(\mu +\gamma +d\right))-\alpha \left(1-c\right)\beta_{1}^{*}S^{0}-\left(1-p\right)\theta \gamma +\left(\mu +\theta \right)\left(\mu +\alpha \right)$.

Therefore, the eigenvalues at the bifurcation point *R*_*eff*_ = 1 is described as; $\lambda_{1}=-\left(\mu +\rho \right)<0$, $\lambda_{2}=\frac{-\left[\left(\mu +\omega \right)+\left(\mu +q\right)\right]-\sqrt{{\left[\left(\mu +\omega \right)+\left(\mu +q\right)\right]}^{2}-4\mu \left(\mu +q+\omega \right)}}{2}=-2\left(\mu +q+\omega \right)<0$, $\lambda_{3}=\frac{-\left[\left(\mu +\omega \right)+\left(\mu +q\right)\right]+\sqrt{{\left[\left(\mu +\omega \right)+\left(\mu +q\right)\right]}^{2}-4\mu \left(\mu +q+\omega \right)}}{2}=-\mu <0$, $\lambda_{4}=0$, $\lambda_{5,6}=\frac{-(\left(\mu +\alpha \right)+\left(\mu +\theta \right)+\left(\mu +\gamma +d\right)-c\beta_{1}^{*}S^{0})}{2}\pm \frac{\sqrt{{\left(\left(\mu +\alpha \right)+\left(\mu +\theta \right)+\left(\mu +\gamma +d\right)-c\beta_{1}^{*}S^{0}\right)}^{2}-4(-((\mu +\alpha )+(\mu +\theta ))(c\beta_{1}^{*}S^{0}-\left(\mu +\gamma +d\right))-\alpha \left(1-c\right)\beta_{1}^{*}S^{0}-\left(1-p\right)\theta \gamma +\left(\mu +\theta \right)\left(\mu +\alpha \right))}}{2}<0$.

Here we observe that;

(i). The Jacobian matrix *J*(*E*^0^) of Eq (5) at the bifurcation parameter such that *β*_1_ = *β*_1_* is denoted by $J(E^{0},\beta_{1}^{*})$, has a single zero eigenvalue with all the other eigenvalues having negative real part.
The Jacobian matrix *J*(*E*^0^) of Eq (5) has a nonnegative right eigenvector **W** and a left eigenvector **V** corresponding to the zero eigenvalue.

Where, $\text{W}={\left(w_{1},w_{2},w_{3},w_{4},w_{5},w_{6}\right)}^{⊺}$ and **V** = (*v*_1_, *v*_2_, *v*_3_, *v*_4_, *v*_5_, *v*_6_), respectively. Then the right eigenvector of the Jacobian matrix $J(E^{0},\beta_{1}^{*})$ becomes:

$$(\begin{array}{cccccc} -(\mu +\omega ) & q & 0 & 0 & 0 & 0\\ \omega & -(\mu +q) & 0 & -\beta_{1}^{*}S^{0} & 0 & \rho \\ 0 & 0 & -(\mu +\alpha ) & \left(1-c\right)\beta_{1}^{*}S^{0} & 0 & 0\\ 0 & 0 & \alpha & c\beta_{1}^{*}S^{0}-\left(\mu +\gamma +d\right) & (1-p)\theta \gamma & 0\\ 0 & 0 & 0 & \gamma & -(\mu +\theta ) & 0\\ 0 & 0 & 0 & 0 & p\theta & -(\mu +\rho ) \end{array})(\begin{array}{c} w_{1}\\ w_{2}\\ w_{3}\\ w_{4}\\ w_{5}\\ w_{6} \end{array})=(\begin{array}{c} 0\\ 0\\ 0\\ 0\\ 0\\ 0 \end{array}).$$

If $D_{1}=\frac{p\rho \theta \gamma }{\left(\mu +\rho )(\mu +\theta \right)}$ and $D_{2}=\frac{\left(\mu +\alpha \right)((\mu +\theta )(\mu +\gamma +d)-(1-p)\theta \gamma )}{\left(\mu +\theta )(\mu c+\alpha \right)}$ then

$$\text{W}=(\begin{array}{c} w_{1}\\ w_{2}\\ w_{3}\\ w_{4}\\ w_{5}\\ w_{6} \end{array})=(\begin{array}{c} \left(\frac{q}{\mu (\mu +q+\omega )}\right)\left(D_{1}-D_{2}\right)w_{4}\\ \left(\frac{\mu +\omega }{\mu (\mu +q+\omega )}\right)\left(D_{1}-D_{2}\right)w_{4}\\ \frac{\left(1-c\right)\beta_{1}^{*}S^{0}}{d(\mu +\alpha )}w_{4}>0\\ w_{4}>0\\ \frac{\gamma }{\mu +\theta }w_{4}>0\\ \frac{p\theta \gamma }{\left(\mu +\rho )(\mu +\theta \right)}w_{4}>0 \end{array}),\;\text{where},\;w_{1},w_{2}>0,\;\text{for}\;D_{1}>D_{2}$$

and the left eigenvectors of the Jacobian matrix $J(E^{0},\beta_{1}^{*})$ becomes:

$$\left(\begin{array}{c} v_{1}\\ v_{2}\\ v_{3}\\ v_{4}\\ v_{5}\\ v_{6} \end{array}{)}^{⊺}(\begin{array}{cccccc} -(\mu +\omega ) & q & 0 & 0 & 0 & 0\\ \omega & -(\mu +q) & 0 & -\beta_{1}^{*}S^{0} & 0 & \rho \\ 0 & 0 & -(\mu +\alpha ) & \left(1-c\right)\beta_{1}^{*}S^{0} & 0 & 0\\ 0 & 0 & \alpha & c\beta_{1}^{*}S^{0}-\left(\mu +\gamma +d\right) & (1-p)\theta \gamma & 0\\ 0 & 0 & 0 & \gamma & -(\mu +\theta ) & 0\\ 0 & 0 & 0 & 0 & p\theta & -(\mu +\rho ) \end{array})=(\begin{array}{c} 0\\ 0\\ 0\\ 0\\ 0\\ 0 \end{array}\right)$$

Hence,

$$\text{V}=(\begin{array}{c} v_{1}\\ v_{2}\\ v_{3}\\ v_{4}\\ v_{5}\\ v_{6} \end{array}{)}^{⊺}=(\begin{array}{c} 0\\ 0\\ \frac{\alpha }{d(\mu +\alpha )}v_{4}>0\\ v_{4}>0\\ \frac{(1-p)\theta }{\left(\mu +\theta \right)}v_{4}>0\\ 0 \end{array}{)}^{⊺}$$

Let *f*_4_ be the 4^*th*^ or the infected component of *F*, i.e.

$$f_{4}=c\eta x_{1}x_{4}+\alpha x_{3}+\left(1-p\right)\theta x_{5}-\left(\mu +\gamma +d\right)x_{4},\;\text{where},\;\eta =(\beta_{1}^{*}-\beta_{2}^{*}\frac{x_{4}}{m+x_{4}}),$$

and the second partial derivative of *f*_4_ is given by:

$$\frac{\partial f_{4}}{\partial x_{4}}=cx_{1}\beta_{1}^{*}-\left(\mu +\gamma +d\right),\;\;\;\frac{\partial^{2}f_{4}}{\partial x_{1}\partial x_{4}}=c\beta_{1}^{*}>0\;\text{and}\;\frac{\partial^{2}f_{4}}{\partial \beta_{1}^{*}\partial x_{4}}=cx_{1}=c\frac{\Lambda }{\mu }(\frac{\pi \mu +\omega }{\mu +q+\omega })>0$$

where, $E^{0}\left(x_{1}^{0},x_{2}^{0},x_{3}^{0},x_{4}^{0},x_{5}^{0},x_{6}^{0}\right)=(\frac{\Lambda }{\mu }(\frac{\pi \mu +\omega }{\mu +q+\omega }),\frac{\Lambda }{\mu }(\frac{\left(q+(1-\pi )\mu \right)}{\left(q+\mu +\omega \right)}),0,0,0,0)$ and $\beta_{1}^{*}=\frac{\mu \left(\mu +\alpha \right)\left(\mu +q+\omega \right)((\mu +\theta )(\mu +\gamma +d)-(1-p)\theta \gamma )}{\Lambda (\mu +\theta )(\mu c+\alpha )(\mu \pi +\omega )}$.

Now we have

$$\begin{array}{c} \begin{array}{cc} a & =\sum_{k,i,j=1}^{n}v_{k}w_{i}w_{j}\frac{\partial^{2}f_{k}}{\partial x_{i}\partial x_{j}}\left(0,0\right)=\sum_{k,i,j=1}^{6}v_{k}w_{2}w_{4}\frac{\partial^{2}f_{4}}{\partial x_{1}\partial x_{4}}\left(0,0\right)\\ & =c\beta_{1}^{*}(\left(\frac{\mu +\omega }{\mu (\mu +q+\omega )}\right)\left(D_{1}-D_{2}\right)w_{4})v_{4}w_{4}\\ b & =\sum_{k,i=1}^{n}v_{k}w_{i}\frac{\partial^{2}f_{k}}{\partial x_{i}\partial \phi }\left(0,0\right)=\sum_{k,i=1}^{6}v_{4}w_{4}\frac{\partial^{2}f_{4}}{\partial x_{4}\partial \beta_{1}}\left(0,0\right)=c\frac{\Lambda }{\mu }(\frac{\pi \mu +\omega }{\mu +q+\omega })v_{4}w_{4}>0 \end{array} \end{array}$$

According to Castillo-Chavez and Song theorem [39] the sign of *a* can be used to determine the direction of the bifurcation.

**Theorem 3.5**. *Using Castillo-Chavez and Song theorem* [39] *if D*_1_ > *D*_2_, *then the backward bifurcation exists at R*_*eff*_ = 1 *in system of model* Eq (1).

**Fig 2:**
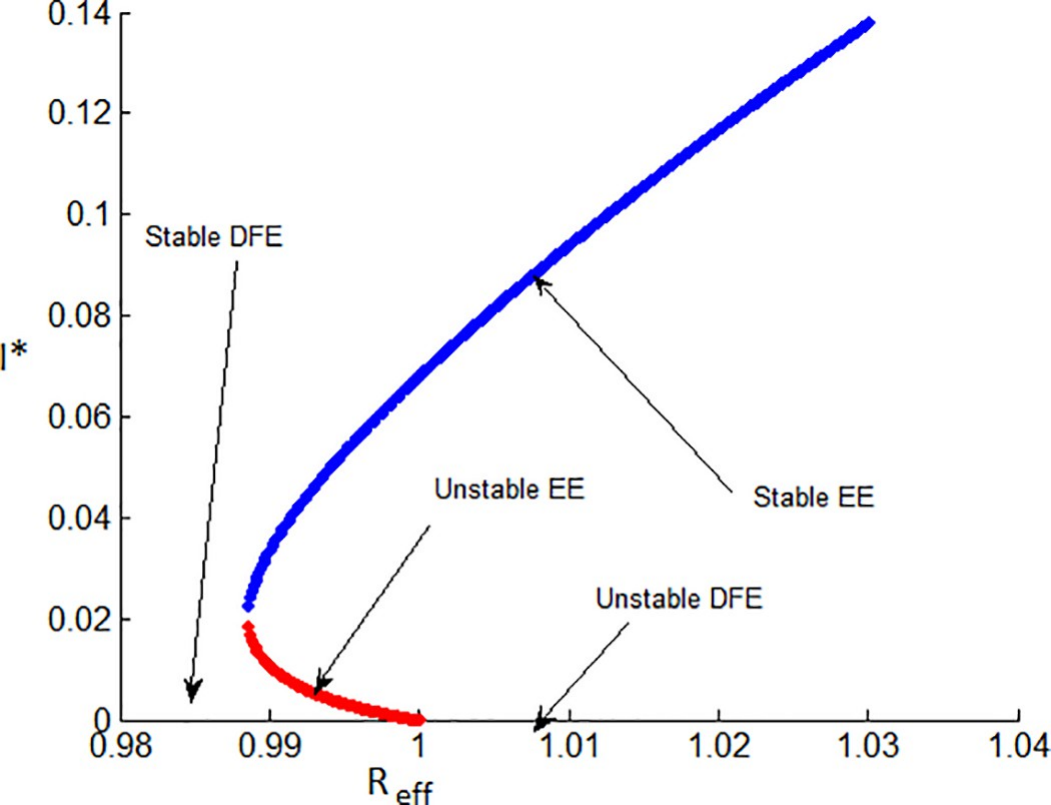
Illustration of backward bifurcation at *R*_*eff*_ = 1.

The diagram in Fig 2 shows the phenomenon of backward bifurcation in the TB disease transmission model (1), where a stable endemic equilibrium co-exists with a stable disease-free equilibrium when the effective reproduction number is less than one. For *R*_*eff*_ < 1, there are two asymptotically stable equilibrium points: these disease-free and endemic equilibrium points. Backward bifurcations have major implications for infectious diseases such as tuberculosis, as control programs based on reducing *R*_*eff*_ below unity may not be effective because the disease can easily persist indefinitely [40]. This indicates that the effective reproduction number is less than one, which is not enough to eliminate TB from society.

## 4 The optimal control problem and analysis

### 4.1 Optimal control problem

Optimal control in epidemiology refers to the use of mathematical and computational optimization techniques to find the best strategies for controlling the spread of infectious diseases within a population [41]. To understand the dynamics under which conditions TB can be controlled or reduced, we apply the concept of optimal control. In this section, we will construct the optimal control problem by incorporating the time-dependent control variables *u*_1_(*t*) and *u*_2_(*t*) for *t* ∈ [0, *T*] and *U* ∈ [0, 1], where *U* = [*u*_1_(*t*), *u*_2_(*t*)]. Here *u*_1_(*t*) represents prevention control (like early diagnosis, case finding, good hygiene, or covering the mouth and nose, media influence and vaccine) and *u*_2_(*t*) represents treatment control (which is provided at home or in hospital). TB infection control is a combination of measures aimed at reducing the risk of TB transmission in populations. The control functions *u*_1_(*t*) and *u*_2_(*t*) are bounded and Lebesgue integrable functions. The coefficient (1 − *u*_1_(*t*)) represents the effectiveness of prevention control strategies aimed at reducing the number of individuals who will become infected. Conversely, (1 + *u*_2_(*t*)) represents efforts to improve strategies, increasing the treatment success rate and decreasing treatment failures. When the control function *u*_1_(*t*) approaches 1, it indicates a high level of prevention efforts, which can effectively minimize the number of infected individual. Similarly, when the control function *u*_2_(*t*) is near 1, it reflects a high rate of successful treatment, contributing to the reduction of the infected population. Then the dynamical system of the model Eq (1) reconstructed with optimal control and it becomes;

$$\begin{array}{c} \begin{array}{cc} \frac{dS}{dt} & =\pi \Lambda -\left(1-u_{1}\right)\eta SI-\left(\mu +q\right)S+\omega V+\rho R,\\ \frac{dV}{dt} & =(1-\pi )\Lambda +qS-(\omega +\mu )V,\\ \frac{dL}{dt} & =\left(1-c\right)\left(1-u_{1}\right)\eta SI-\left(\mu +\alpha \right)L,\\ \frac{dI}{dt} & =c\left(1-u_{1}\right)\eta SI+\alpha L+\left(1-p\right)\theta T-\left(\mu +\left(1+u_{2}\right)\gamma +d\right)I,\\ \frac{dT}{dt} & =\left(1+u_{2}\right)\gamma I-\left(\theta +\mu \right)T,\\ \frac{dR}{dt} & =p\theta T-(\mu +\rho )R, \end{array} \end{array}$$ (9)

with the initial condition: *S*_0_ ≥ 0, *V*_0_ ≥ 0, *L*_0_ ≥ 0, *I*_0_ ≥ 0, *T*_0_ ≥ 0, *R*_0_ ≥ 0, and $\eta =(\beta_{1}-\beta_{2}\frac{I}{m+I})$.

In our work we want to minimized the latent TB individual and the TB infected individuals with constant coefficient *A*_1_ and *A*_2_. While the constant coefficient *B*_1_ and *B*_2_ are balancing cost factor due to size and importance of objective functional. The aim of this section is to minimize the functional objectives of the problem (9). Then our functional objective to be minimized is constructed as in Eq (10):

$$\begin{array}{c} J\left(u_{1},u_{2}\right)=\int_{0}^{T}(A_{1}L\left(t\right)+A_{2}I\left(t\right)+\frac{B_{1}}{2}u_{1}^{2}\left(t\right)+\frac{B_{2}}{2}u_{2}^{2}\left(t\right))dt \end{array}$$ (10)

In this work, our goal is to find an optimal control *u*_1_(*t*)* and *u*_2_(*t*)* by using Pontryagin’s minimum principle theorem in reference [41]. This implies that;

$$\begin{array}{c} \begin{array}{cc} J(u_{1}{\left(t\right)}^{*},u_{2}{\left(t\right)}^{*}) & =\underset{\Omega_{u}}{\text{min}}\;J(u_{1}\left(t\right),u_{2}\left(t\right))\\ & \;\text{subject}\;\text{to}\;\text{;}\;(10) \end{array} \end{array}$$ (11)

Where, Ω = {(*u*_1_(*t*), *u*_2_(*t*)) ∈ *L*^2^(0, *T*) | 0 ≤ *u*_1_, *u*_2_ ≤ 1}, and the state variables are (*S*, *V*, *L*, *I*, *T*, *R*) with initial condition, *V*_0_ ≥ 0, *L*_0_ ≥ 0, *I*_0_ ≥ 0, *T*_0_ ≥ 0, *R*_0_ ≥ 0.

### 4.2 Existence of an optimal control

**Theorem 4.1**. *There exists an optimal solutions of problem* (9) $U^{*}=\left(u_{1}^{*},u_{2}^{*}\right)$ and the corresponding solution of state functions X* = (S*, V*, L*, I*, T*, R*) *that minimizes J*(*u*_1_, *u*_2_) *in the optimal control problem* (*12*). *Let U*(*t*) = (*u*_1_, *u*_2_) *be a time optimal control and X*(*t*) = (*S*, *V*, *L*, *I*, *T*, *R*) *be the corresponding response of the system. Then the Optimal Control Problem is given by*:

$$\begin{array}{c} \begin{array}{cc} \;\underset{U\in \Omega_{u}}{\text{min}} & \int_{0}^{T}(A_{1}L\left(t\right)+A_{2}I\left(t\right)+\frac{B_{1}}{2}u_{1}^{2}\left(t\right)+\frac{B_{2}}{2}u_{2}^{2}\left(t\right))dt\\ s.t & \;\;\dot{X}=\Phi \left(X\left(t\right),U\left(t\right),t\right)=\left(10\right)\\ \;\; & X\left(0\right)=X_{0}=\left(S_{0}\geq 0,V_{0}\geq 0,L_{0}\geq 0,I_{0}\geq 0,T_{0}\geq 0,R_{0}\geq 0\right)\\ \;\; & U(t)\in [0,1],\;\;\;\;\;0\leq t\leq T \end{array} \end{array}$$ (12)

*Proof*. Pontryagin’s minimum principle states that the optimal state trajectory X*, optimal control U*, and corresponding Lagrange multiplier vector λ* must minimize the Hamiltonian *H* such that,

$$\begin{array}{c} H\left(t,X^{*}\left(t\right),U^{*}\left(t\right),\lambda^{*}\left(t\right)\right)=\underset{U\in [0,1]}{\text{min}}\left(H\left(t,X\left(t\right),U\left(t\right),\lambda \left(t\right)\right)\right). \end{array}$$ (13)

For all controls *U* at time *t* the Hamiltonian equation *H* is given by:

$$\begin{array}{c} H(X(t),U(t),\lambda (t),t)=f(X(t),U(t),t)-\lambda (t)\Phi (X(t),U(t),t). \end{array}$$ (14)

If U*(*t*) is solutions of an optimal control and X*(*t*) is corresponding optimal trajectory of the above optimal control problem (13), then there is a costate function λ*(*t*) such that the followings hold:

1. Pontryagin’s minimum principle, U* is the minimizer of *H*(X*(*t*), *U*(*t*), λ*(*t*), *t*) over *U*.
2. X*(*t*) and λ*(*t*) are solutions of:
  Adjoint Equation(ADJ)     $\dot{\lambda }=H_{X}\left(X,U^{*},\lambda ,t\right)$
  Transversality Condition(TR)     λ(*T*) = 0
  State Equation (ODE)     $\dot{X}=\phi \left(X,U,t\right)$
  Initial Condition(IC)     *X*(0) = *X*_0_ = (*S*_0_ ≥ 0, *V*_0_ ≥ 0, *L*_0_ ≥ 0, *I*_0_ ≥ 0, *T*_0_ ≥ 0, *R*_0_ ≥ 0)

Now the Hamiltonian equation is given by:

$$\begin{array}{c} \begin{array}{cc} H(X(t),U(t),\lambda (t),t) & =f(X(t),U(t),t)-\lambda (t)\phi (X(t),U(t),t)\\ & =A_{1}L\left(t\right)+A_{2}I\left(t\right)+\frac{B_{1}}{2}u_{1}^{2}\left(t\right)+\frac{B_{2}}{2}u_{2}^{2}\left(t\right)-\sum_{i=1}^{6}\lambda_{i}\left(t\right)\phi_{i} \end{array} \end{array}$$

where, *ϕ*_*i*_ represents the right hand side Eq (10) and λ_*i*_(*t*) is adjoint function for *i* = 1, …, 6.

$$\begin{array}{c} \begin{array}{cc} =A_{1}L\left(t\right)+A_{2}I\left(t\right)+\frac{B_{1}}{2}u_{1}^{2}\left(t\right)+\frac{B_{2}}{2}u_{2}^{2}\left(t\right)- & \lambda_{1}\left(t\right)(\pi \Lambda -\left(1-u_{1}\right)\varphi_{1}SI-\left(\mu +q\right)S+\omega V+\rho R)\\ - & \lambda_{2}\left(t\right)((1-\pi )\Lambda +qS-(\omega +\mu )V)\\ - & \lambda_{3}\left(t\right)(\left(1-c\right)\left(1-u_{1}\right)\varphi_{1}SI-\left(\mu +\alpha \right)L)\\ - & \lambda_{4}\left(t\right)(c\left(1-u_{1}\right)\varphi_{1}SI+\alpha L+\left(1-p\right)\theta T-\left(\mu +\left(1+u_{2}\right)\gamma +d\right)I)\\ - & \lambda_{5}\left(t\right)(\left(1+u_{2}\right)\gamma I-\left(\theta +\mu \right)T)\\ - & \lambda_{6}\left(t\right)(p\theta T-(\mu +\rho )R) \end{array} \end{array}$$

To get the optimal control solution, we need to find the partial derivative of the Hamiltonian equation with respect to the control variable (condition 1) becomes:

$$\begin{array}{c} \begin{array}{cc} \frac{\partial H}{\partial u_{1}} & =B_{1}u_{1}\left(t\right)-\lambda_{1}\left(t\right)\varphi_{1}SI+\lambda_{3}\left(t\right)\left(1-c\right)\varphi_{1}SI+\lambda_{4}\left(t\right)c\varphi_{1}SI=0\\ & \Rightarrow {\hat{u}}_{1}\left(t\right)=\frac{\left(\lambda_{1}\left(t\right)-\lambda_{3}\left(t\right)\left(1-c\right)-\lambda_{4}\left(t\right)c\right)}{B_{1}}\varphi_{1}SI\\ \frac{\partial H}{\partial u_{2}} & =B_{2}u_{2}\left(t\right)+\lambda_{4}\left(t\right)\gamma I-\lambda_{5}\left(t\right)\gamma I=0\\ & \Rightarrow {\hat{u}}_{2}\left(t\right)=\frac{\left(\lambda_{5}\left(t\right)-\lambda_{4}\left(t\right)\right)}{B_{2}}\gamma I \end{array} \end{array}$$

Now there exists an adjoint function λ_1_(*t*), …, λ_6_(*t*), such that;

$$\begin{array}{c} \begin{array}{cc} {\dot{\lambda }}_{1}\left(t\right)=H_{S} & =\lambda_{1}\left(t\right)(\left(1-u_{1}\right)\varphi_{1}I+\left(\mu +q\right))-\lambda_{2}\left(t\right)q-\lambda_{3}\left(t\right)\left(1-c\right)\left(1-u_{1}\right)\varphi_{1}I-\lambda_{4}\left(t\right)c\left(1-u_{1}\right)\varphi_{1}I\\ & =(\lambda_{1}\left(t\right)-\left(1-c\right)\lambda_{3}\left(t\right)-c\lambda_{4}\left(t\right))\left(1-u_{1}\right)\varphi_{1}I-q\lambda_{2}\left(t\right)+\left(\mu +q\right)\lambda_{1}\left(t\right)\\ {\dot{\lambda }}_{2}\left(t\right)=H_{V} & =-\omega \lambda_{1}\left(t\right)+\left(\omega +\mu \right)\lambda_{2}\left(t\right)\\ {\dot{\lambda }}_{3}\left(t\right)=H_{L} & =A_{1}+\left(\mu +\alpha \right)\lambda_{3}\left(t\right)-\alpha \lambda_{4}\left(t\right)\\ {\dot{\lambda }}_{4}\left(t\right)=H_{I} & =A_{2}+\frac{\partial (\lambda_{1}\left(t\right)-\lambda_{3}\left(t\right)\left(1-c\right)-\lambda_{4}\left(t\right)c)\left(1-u_{1}\right)\varphi_{1}SI}{\partial I}+\lambda_{4}\left(t\right)\left(\mu +\left(1+u_{2}\right)\gamma +d\right)-\lambda_{5}\left(1+u_{2}\right)\gamma \\ & =A_{2}+(\lambda_{1}\left(t\right)-\left(1-c\right)\lambda_{3}\left(t\right)-c\lambda_{4}\left(t\right))\left(1-u_{1}\right)S(\frac{\partial (\varphi_{1}I)}{\partial I})+\left(\mu +\left(1+u_{2}\right)\gamma +d\right)\lambda_{4}\left(t\right)\\ & -\left(1+u_{2}\right)\gamma \lambda_{5}\;\text{where,}\;\frac{\partial (\varphi_{1}I)}{\partial I}=\frac{\beta_{1}m^{2}+2m\left(\beta_{1}-\beta_{2}\right)I+\left(\beta_{1}-\beta_{2}\right)I^{2}}{{\left(m+I\right)}^{2}}\\ {\dot{\lambda }}_{5}\left(t\right)=H_{T} & =-\left(1-p\right)\theta \lambda_{4}\left(t\right)+\left(\mu +\theta \right)\lambda_{5}\left(t\right)-p\theta \lambda_{6}\left(t\right)\\ {\dot{\lambda }}_{6}\left(t\right)=H_{R} & =-\rho \lambda_{1}\left(t\right)+\left(\mu +\rho \right)\lambda_{6}\left(t\right) \end{array} \end{array}$$ (15)

with transversality conditions,

$$\begin{array}{c} \begin{array}{cc} \lambda_{1}\left(T\right)= & 0,\lambda_{2}\left(T\right)=0,\lambda_{3}\left(T\right)=0,\lambda_{4}\left(T\right)=0,\lambda_{5}\left(T\right)=0,\lambda_{6}\left(T\right)=0. \end{array} \end{array}$$ (16)

Furthermore, we may characterize the associated optimal functions $u_{1}^{*}\left(t\right),u_{2}^{*}\left(t\right)$ by the following functions

$$\begin{array}{c} \begin{array}{cc} {\hat{u}}_{1}\left(t\right)= & \frac{\left(\lambda_{1}\left(t\right)-\left(1-c\right)\lambda_{3}\left(t\right)-c\lambda_{4}\left(t\right)\right)}{A_{3}}\varphi_{1}S^{*}I^{*}\\ {\hat{u}}_{2}\left(t\right)= & \frac{\left(\lambda_{5}\left(t\right)-\lambda_{4}\left(t\right)\right)}{A_{4}}\gamma I^{*} \end{array} \end{array}$$

Then

$$\begin{array}{c} u_{1}^{*}=\{\begin{array}{c} 0,\;\text{if}\;{\hat{u}}_{1}<0\\ {\hat{u}}_{1},\;\text{if}\;0\leq {\hat{u}}_{1}<1\\ 1,\;\text{if}\;{\hat{u}}_{1}>1 \end{array}\;\text{and}\;\;u_{2}^{*}=\{\begin{array}{c} 0,\;\text{if}\;{\hat{u}}_{2}<0\\ {\hat{u}}_{2},\;\text{if}\;0\leq {\hat{u}}_{2}<1\\ 1,\;\text{if}\;{\hat{u}}_{2}>1 \end{array} \end{array}$$

Therefore,

$$\begin{array}{c} \begin{array}{cc} u_{1}^{*}= & \text{min}\{\text{max}\{0,\frac{\left(\lambda_{1}\left(t\right)-\left(1-c\right)\lambda_{3}\left(t\right)-c\lambda_{4}\left(t\right)\right)}{B_{1}}\varphi_{1}S^{*}I^{*}\},1\}\\ u_{2}^{*}= & \text{min}\{\text{max}\{0,\frac{\left(\lambda_{5}\left(t\right)-\lambda_{4}\left(t\right)\right)}{B_{2}}\gamma I^{*}\},1\} \end{array} \end{array}$$ (17)

Hence, the theorem.

## 5 Sensitivity analysis

In this section we perform a sensitivity analysis of both the basic and effective reproduction number. Sensitivity analysis of the reproduction number is conducted to find parameters of the model that are most sensitive and should be targeted by intervention strategies. Sensitivity analysis is often used to determine the robustness of model predictions to parameter values [42]. A parameter is called sensitive if small changes in its value produce large changes in the solution of the TB model (1). Definition 1 is used to find the sensitivity index of each of the parameters involved in *R*_0_ and *R*_*eff*_.

**Definition 1**. *The normalized forward sensitivity index of the reproduction numbers R*_*eff*_ *and R*_0_, *denoted by* (*R*_0_)_*P*_ *and* (*R*_*eff*_)_*P*_, *respectively, is defined as*

$$\begin{array}{c} (R_{eff}{)}_{P}=\frac{\partial R_{eff}}{\partial P}\times \frac{P}{R_{eff}}\;\text{and}\;(R_{0}{)}_{P}=\frac{\partial R_{0}}{\partial P}\times \frac{P}{R_{0}}. \end{array}$$ (18)

*Then, the most sensitive parameter is the one with the highest magnitude as compared to others*.

Thus the sensitivity index of *R*_*eff*_ with respect to *P*, where *P* ∈ (*β*_1_, Λ, *ω*, *γ*, *θ*, *α*, *ρ*, *π*, *p*, *c*, *d*, *q*) is computed at Table 3.

**Table 3:** Sensitivity index of effective reproduction number with respect to each parameters.

Parameters	Sensitivity index of *R*_*eff*_	Sensitivity value
*β*_1_	1	1
Λ	1	1
*ω*	$\frac{\mu \omega (1-\pi )+q\omega }{\left(\mu +q+\omega )(\mu \pi +\omega \right)}$	0.56342
*γ*	$\frac{-\gamma (\mu +P\theta )}{(\mu +\gamma +d)(\mu +\theta )-(1-P)\theta \gamma }$	−0.766024
*θ*	$\frac{\mu (1-P)\theta \gamma }{\left(\mu +\theta )((\mu +\gamma +d)(\mu +\theta )-(1-P)\theta \gamma \right)}$	0.004655
*α*	$\frac{(1-c)\alpha \mu }{\left(\mu +\alpha )(\mu c+\alpha \right)}$	0.31746
*q*	$\frac{-q}{\left(\mu +q+\omega \right)}$	−0.58083
*d*	$\frac{-d(\mu +\theta )}{\left(\mu +\gamma +d)(\mu +\theta )-(1-P)\theta \gamma \right)}$	−0.20645
*c*	$\frac{c\mu }{\mu c+\alpha }$	0.4444
*π*	$\frac{\pi \mu }{\mu \pi +\omega }$	0.03041
*p*	$\frac{-P\theta \gamma }{(\mu +\gamma +d)(\mu +\theta )-(1-P)\theta \gamma }$	−0.24902

The sensitivity index of *R*_0_ with respect to *P*, where *P* ∈ (*β*_1_, Λ, *γ*, *θ*, *α*, *ρ*, *π*, *p*, *c*, *d*, *μ*) are computed at Table 4. The basic reproduction number (*R*_0_) is defined by assuming the TB mathematical model (1) without vaccine.

**Table 4:** Sensitivity index of basic reproduction number with respect to each parameters.

Parameters	Sensitivity index of *R*_0_	Sensitivity value
*β*_1_	1	1
Λ	1	1
*γ*	$\frac{-\gamma (\mu +p\theta )}{(\mu +\gamma +d)(\mu +\theta )-(1-P)\theta \gamma }$	−0.766024
*θ*	$\frac{\mu (1-p)\theta \gamma }{\left(\mu +\theta )((\mu +\gamma +d)(\mu +\theta )-(1-P)\theta \gamma \right)}$	0.004655
*α*	$\frac{(1-c)\alpha \mu }{\left(\mu +\alpha )(\mu c+\alpha \right)}$	0.31746
*d*	$\frac{-d(\mu +\theta )}{\left(\mu +\gamma +d)(\mu +\theta )-(1-p)\theta \gamma \right)}$	−0.20645
*c*	$\frac{c\mu }{\mu c+\alpha }$	0.4444
*π*	1	1
*p*	$\frac{-p\theta \gamma }{(\mu +\gamma +d)(\mu +\theta )-(1-p)\theta \gamma }$	−0.24902

Where the parameter values are listed in Table 5.

**Table 5:** Parameter values of the mathematical model (1).

Parameters	Value per year	Reference
Λ	250	[43]
*q*	0.715	[35]
*ω*	0.5	[35]
*μ*	0.016	[18]
*d*	0.12	[4]
*β*_1_	0.0001 − 0.09	Assumed
*β*_2_	0.00005 − 0.01	Assumed
*m*	40, 100	[11]
*α*	0.005	[20]
*γ*	0.59	[4]
*θ*	0.84	[4]
*ρ*	0.05	[11]
*π*	0.98	[3]
*c*	0.25	[11]
*p*	0.75	Assumed

A sensitivity index with positive or negative sign shows that the parameter has direct or indirect effect on the reproduction number [20]. Which means if the sensitivity index result is negative, then the relationship between the parameters and *R*_*eff*_ or *R*_0_ is inversely proportional, and if it is positive, then it is directly proportional. From the sensitivity index that is calculated at Tables 3 and 4, we observe that the parameters *γ*, *q*, *p* & *d* have negative signs. This means an increase (decrease) of these parameters, then the value of *R*_0_ and *R*_*eff*_ will decrease (increase), which has inversely proportional [44]. From the fact that when treatment rate (*γ*) and proportion of the recovery rate (*p*) of TB disease increases, the reproduction number decreases because TB cases under treatment cannot be infectious. When the TB vaccine rate (*q*) also increases, the reproduction number decreases. From Tables 3 & 4, we observe that the values of *S*_*β*_ and *S*_Λ_ are exactly +1; this means that an increase in *β* and Λ will lead to an increase in *R*_0_ and *R*_*eff*_ in the same proportion. The remaining parameter indices *ω*, *α* & *θ* have a positive sign, and this indicates also a decrease (increase) in these parameters and a decrease (increase) in the reproduction number. The most sensitive parameter has a magnitude of the sensitivity index larger than that of all other parameters [35]. From Figs 4 & 5, and Tables 3 & 4 we observe the most sensitive parameters of the mathematical model (1). Hence the most sensitive parameters are the transmission rate of TB (*β*_1_) and the recruitment rate (Λ). The treatment rate (*γ*), vaccination rate of susceptible (*q*), and vaccine waning rate (*ω*), are also the most sensitive parameters next to the two parameters.

If the treatment rate (*γ*) varies, the sensitivity index of the parameters *γ* varies, which shows that treatment rate *γ* is the most sensitive parameter. If the vaccine rate (*q*) varies, the sensitivity index of the parameters *q* varies, which shows that vaccination rate *q* is the most sensitive parameter as shown in Table 6.

**Table 6:** Sensitivity index value when treatment and vaccine rate varies.

Parameters	Parameter value varies	Sensitivity index value
*γ*	for *γ* = 0.25	-0.2033
*γ*	for *γ* = 0.59	-0.766024
*γ*	for *γ* = 0.75	-0.82207
*q*	for *q* = 0.25	-0.32637
*q*	for *q* = 0.715	-0.58083
*q*	for *q* = 0.9	-0.63559

Using the values of the parameters in Table 5, we computed both the effective and basic reproduction numbers. These are *R*_0_ = 10.6 and *R*_*eff*_ = 4.3, which means the TB disease is spread in the community. Here we note that the effects of vaccination have a significant role in reducing the spread of TB. Since the effective reproduction number is reduced by more than half of the basic reproduction number, this means that the basic reproduction number is calculated without vaccination parameters.

## 6 Numerical results and discussion

This section presents numerical simulations using MATLAB’s ode45 to analyze the effects of control measures on TB transmission based on the discussed mathematical models. The simulations focus on the impacts of two control variables (*u*_1_ and *u*_2_) and their combination on the effective reproduction number and state equations. Prevention controls include measures like covering the mouth when coughing and maintaining hygiene, while treatment controls involve medical interventions for infected individuals. Using initial population values (*S*(0) = 2000, *V*(0) = 500, *L*(0) = 1000, *I*(0) = 100, *T*(0) = 50, *andR*(0) = 20), the numerical simulation aims to illustrate the system’s behavior under these controls.

### 6.1 Discussions

**Fig 3:**
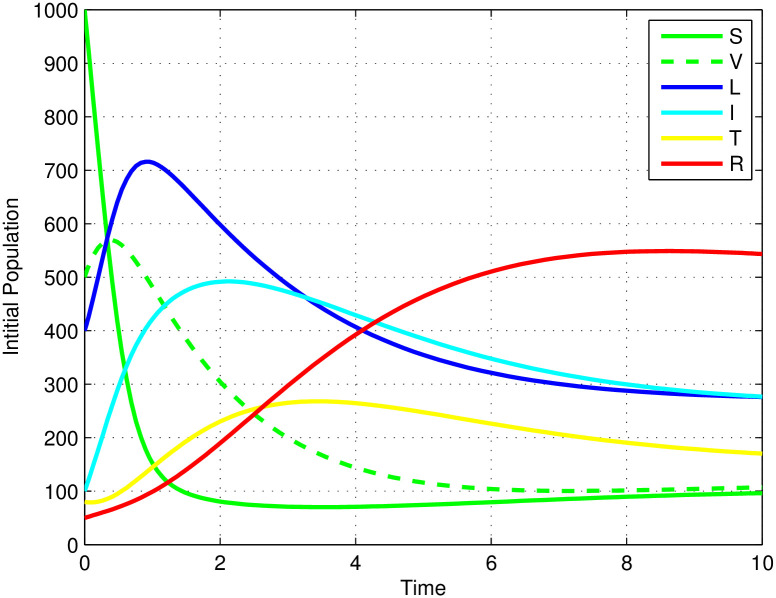
The solution behavior of the mathematical model of the TB dynamical system (1) when *R*_*eff*_ = 7.3662 > 1.

**Fig 4:**
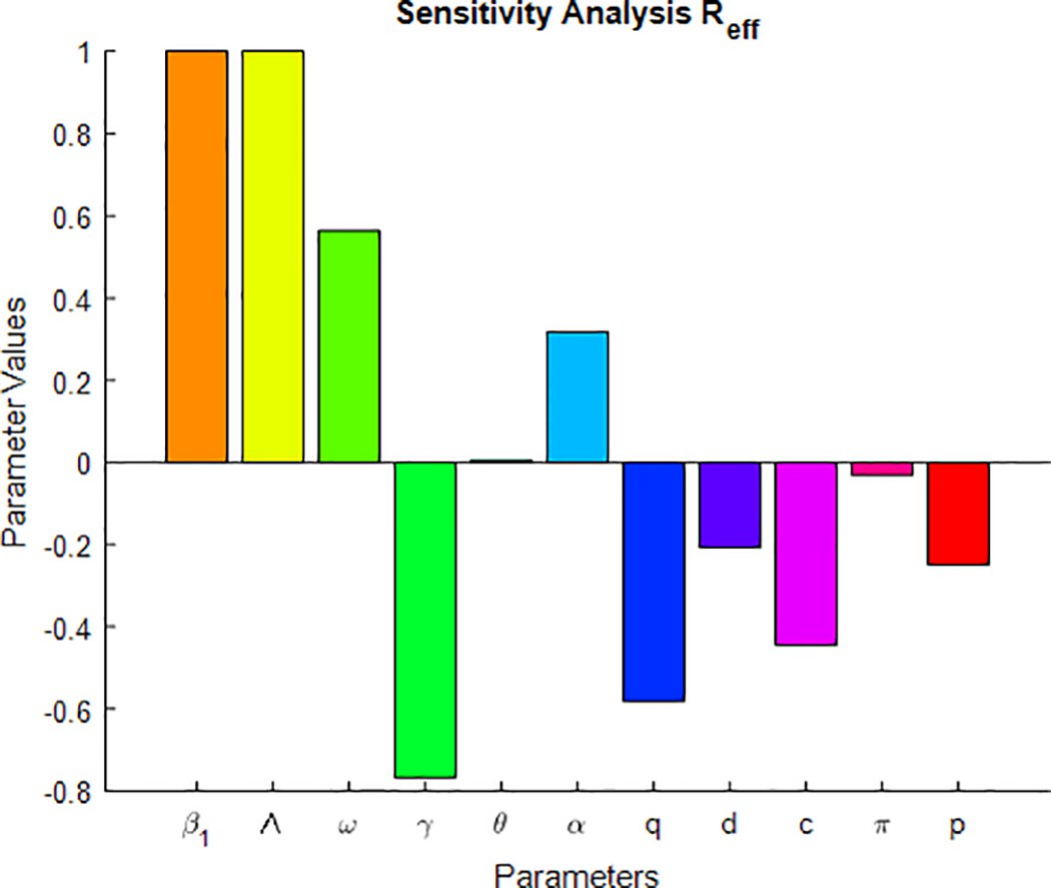
Sensitivity indices diagram for *R*_*eff*_.

**Fig 5:**
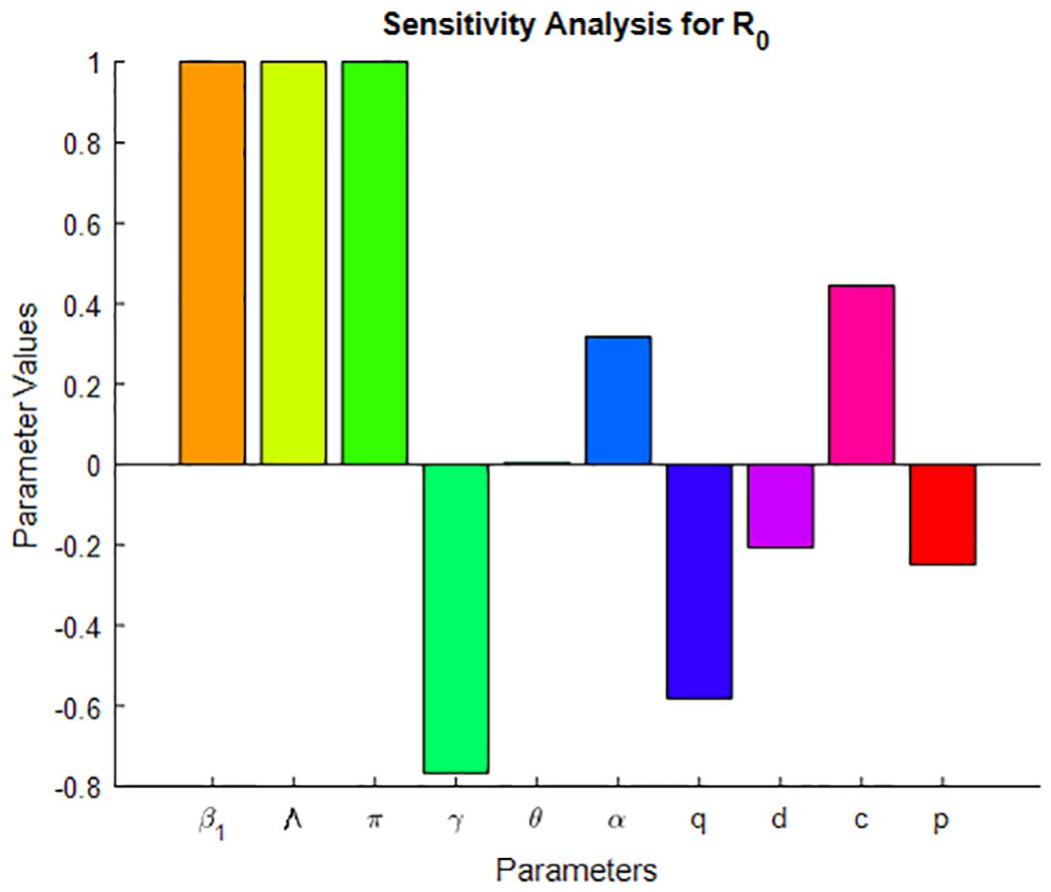
Sensitivity indices diagram for *R*_0_.

Fig 3 illustrates the stability of the local endemic equilibrium in an infectious disease model. It shows a decline in latent TB cases as they progress to active TB, while active cases decrease due to treatment. The number of treated individuals is also declining because of re-infections and recovery. As treated individuals recover, the number of recovered individuals increases. A sensitivity index identifies the most influential parameters affecting the basic (*R*_0_) and effective (*R*_*eff*_) reproduction numbers, as detailed in Figs 4 and 5. Parameters such as Λ, *β*_1_, *ω*, *γ*, *θ*, *α*, *q*, *d*, *c*, and *p* can significantly impact both *R*_0_ and *R*_*eff*_, leading to their increase or decrease.

**Fig 6:**
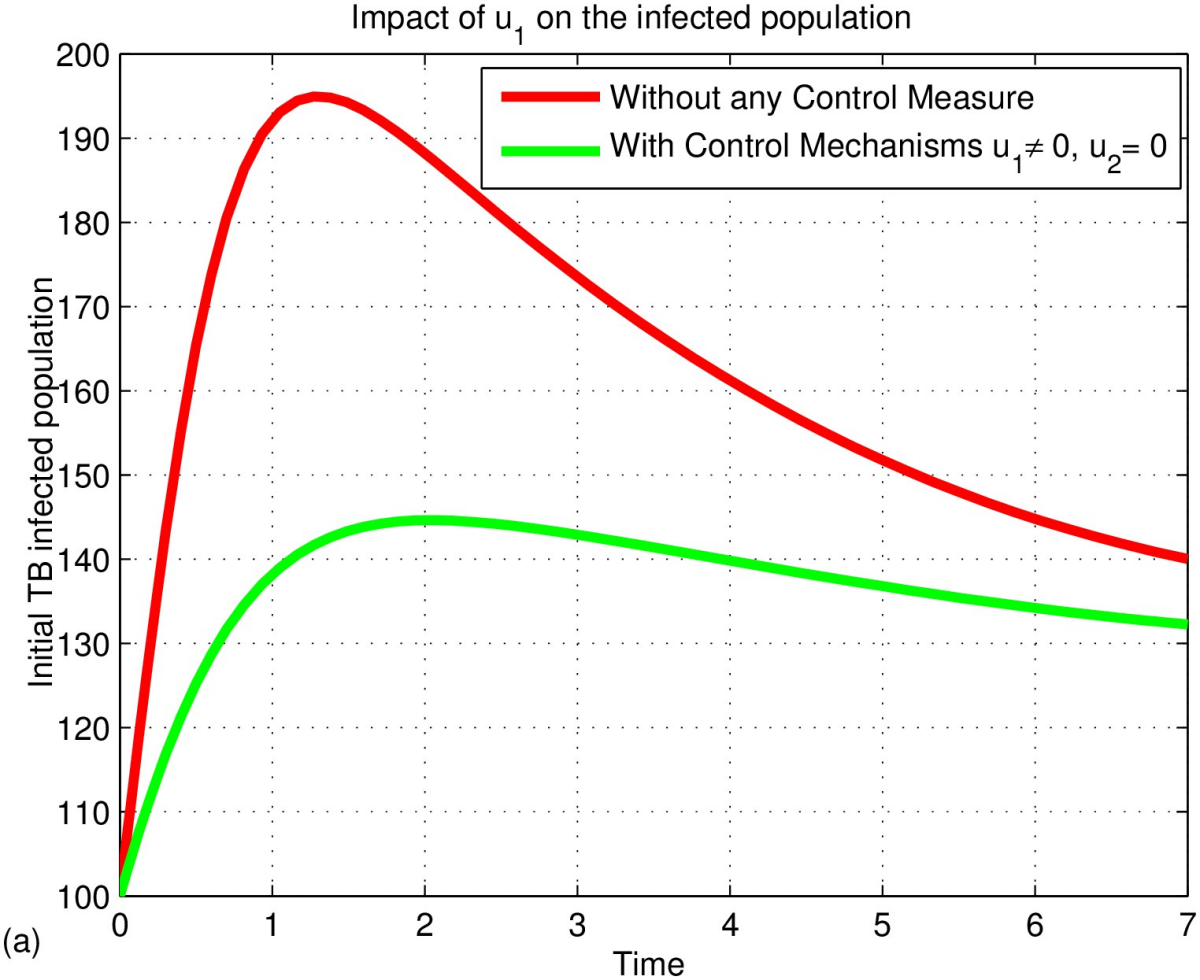
Impact of *u*_1_ on the infected populations.

**Fig 7:**
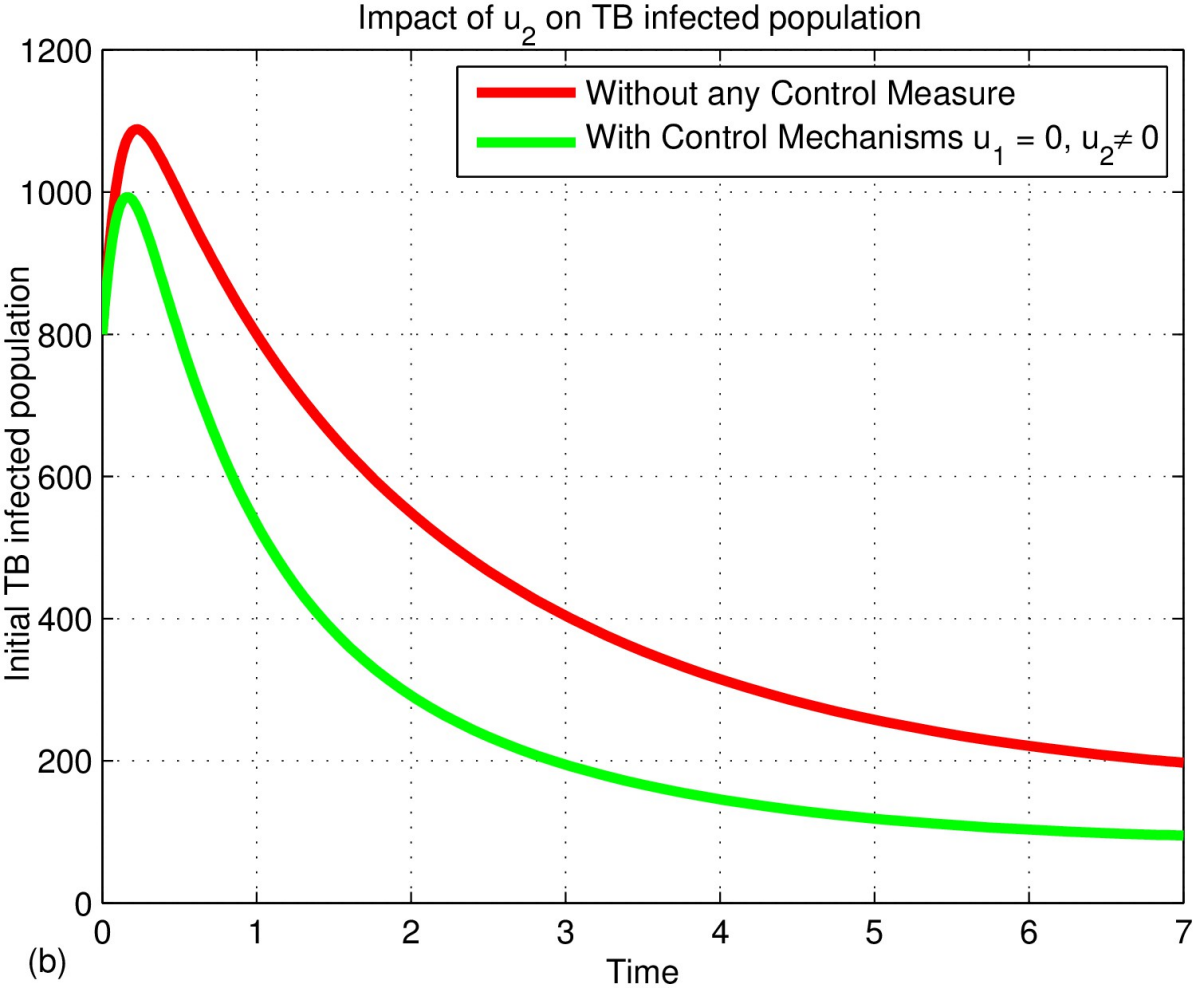
Impact of *u*_2_ on the infected populations.

**Fig 8:**
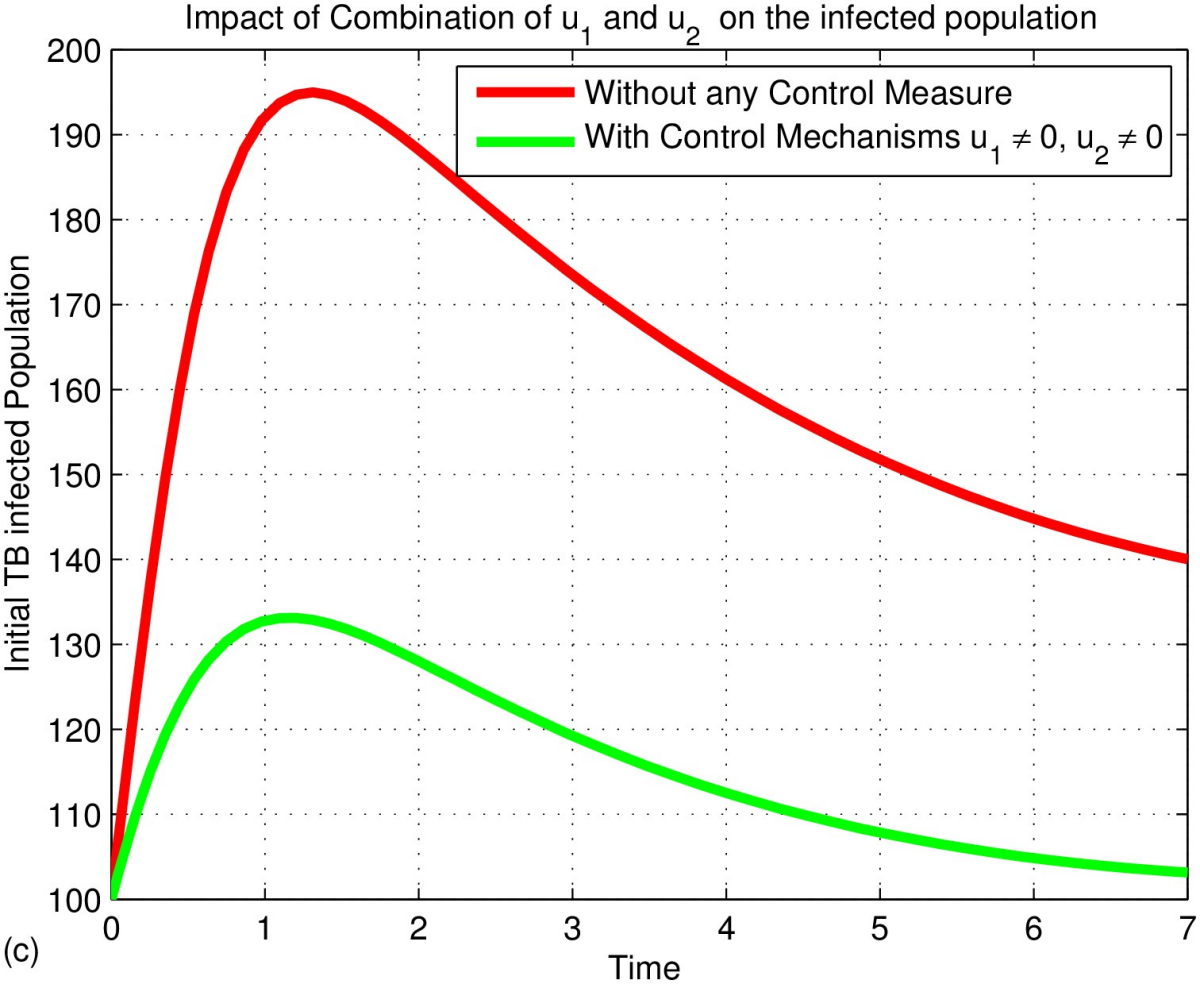
Simulation of the impact of various control strategies on the TB infected population.

Figs 6–8 illustrates the dynamics of the TB-infected population with and without control strategies. Fig 6 demonstrates that the prevention control strategy (*u*_1_) significantly reduces the number of infected individuals compared to no control. Fig 7 shows that the treatment control (*u*_2_) also has a substantial impact in lowering TB cases. In Fig 8, the combination of both controls (*u*_1_ and *u*_2_) proves to be the most effective in reducing TB incidence. The simulation indicates that prevention (*u*_1_) and treatment (*u*_2_) are key strategies in eradicating TB from the community. In fact, TB prevention protects both the individual and the community from TB disease by reducing the transmission of TB from infected people to the susceptible people. In addition, WHO supports countries in preventing TB infection through guidelines and implementation of infection prevention measures. These measures are critical in areas where the risk of TB transmission is high, such as health care facilities, assembly areas, and areas where TB-affected families are present. The risk of developing active TB disease is much higher for people with weakened immune systems or people with diabetes, cancer, or HIV; that is why TB prevention is more important for those people. The TB vaccine (BCG) is one of the TB prevention treatments that prevents children from contracting severe TB. The economic impact of tuberculosis comes from the scale of the problem, and in developing countries, most disease and death occur among the most economically active segments of the population. From this point of view, the preventive measures we take before contracting TB disease will not have much economic impact as they go along with our daily lives. However, the treatments we take after being diagnosed with TB have a significant economic impact. Because there will be drug costs, the patient’s work will stop, and the person who helps the patient will lose his job. As a result, labor is misused and production power is reduced, so it is known to cause economic crises. This will have an economic, social, and logistical impact on society. Therefore, TB prevention is a better control strategy than TB treatment. However, since not everyone can prevent TB, if people affected by the disease go to a medical facility in time and receive treatment and follow up properly, they can at least prevent the disease from being transmitted to other people. Therefore, if they prevent TB disease in advance and treat it after it is caught, the spread of the TB disease will be reduced. Therefore, we can conclude that the two controls, namely prevention (*u*_1_) and treatment (*u*_2_), play an important role in eradicating TB from the community. As we can see from Fig 8, it indicates that prevention and treatment control strategies have an important role in reducing the spread of TB disease. This means that if the individuals who are not infected with the TB disease can be careful not to get TB, if the infected individuals follow their treatment and take their medicine properly, the combination of the two controls will help to prevent the spreading of TB disease to other people and prevent drug-resistant TB.

**Fig 9:**
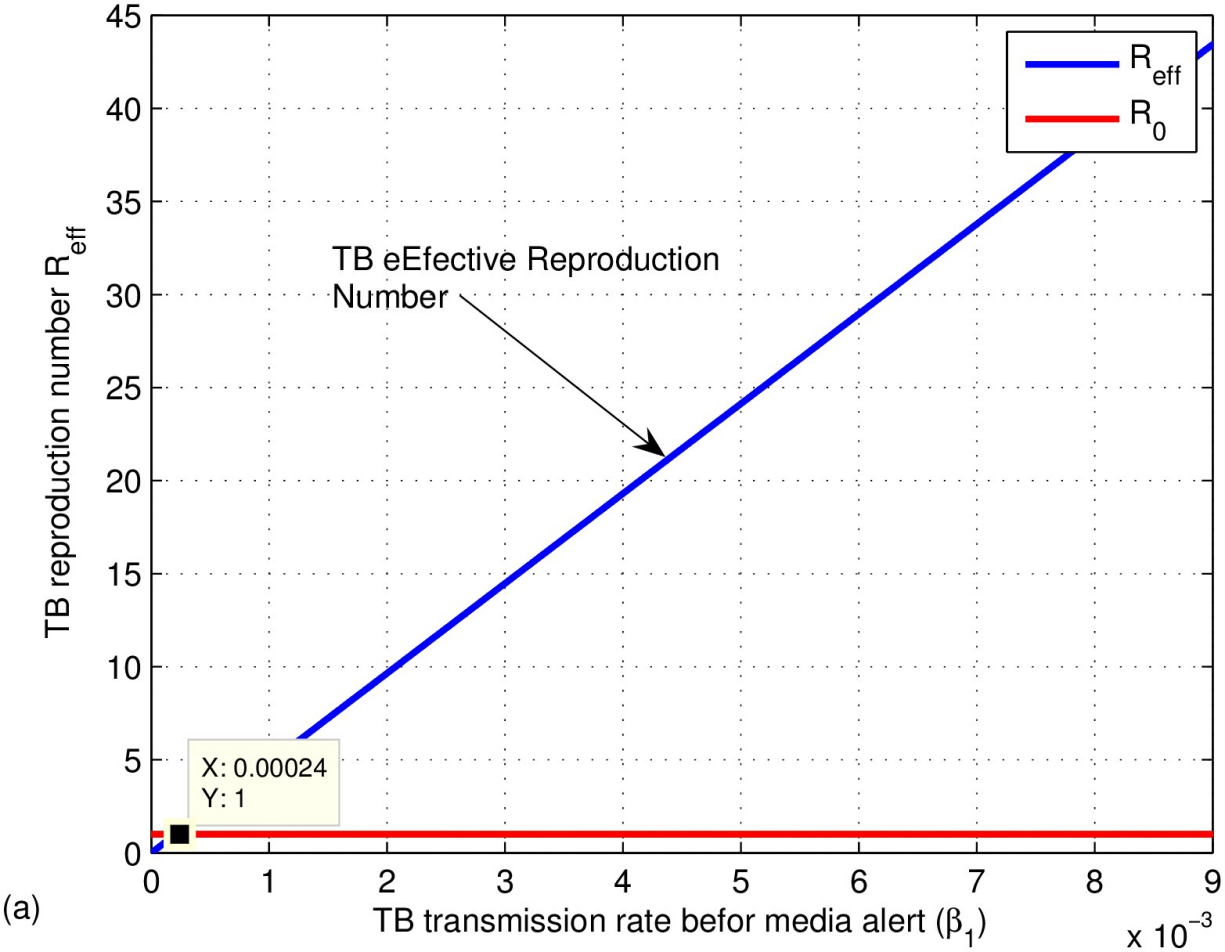
Effect of transmission rate before media alert (*β*_1_) on the effective reproduction number *R*_*eff*_.

**Fig 10:**
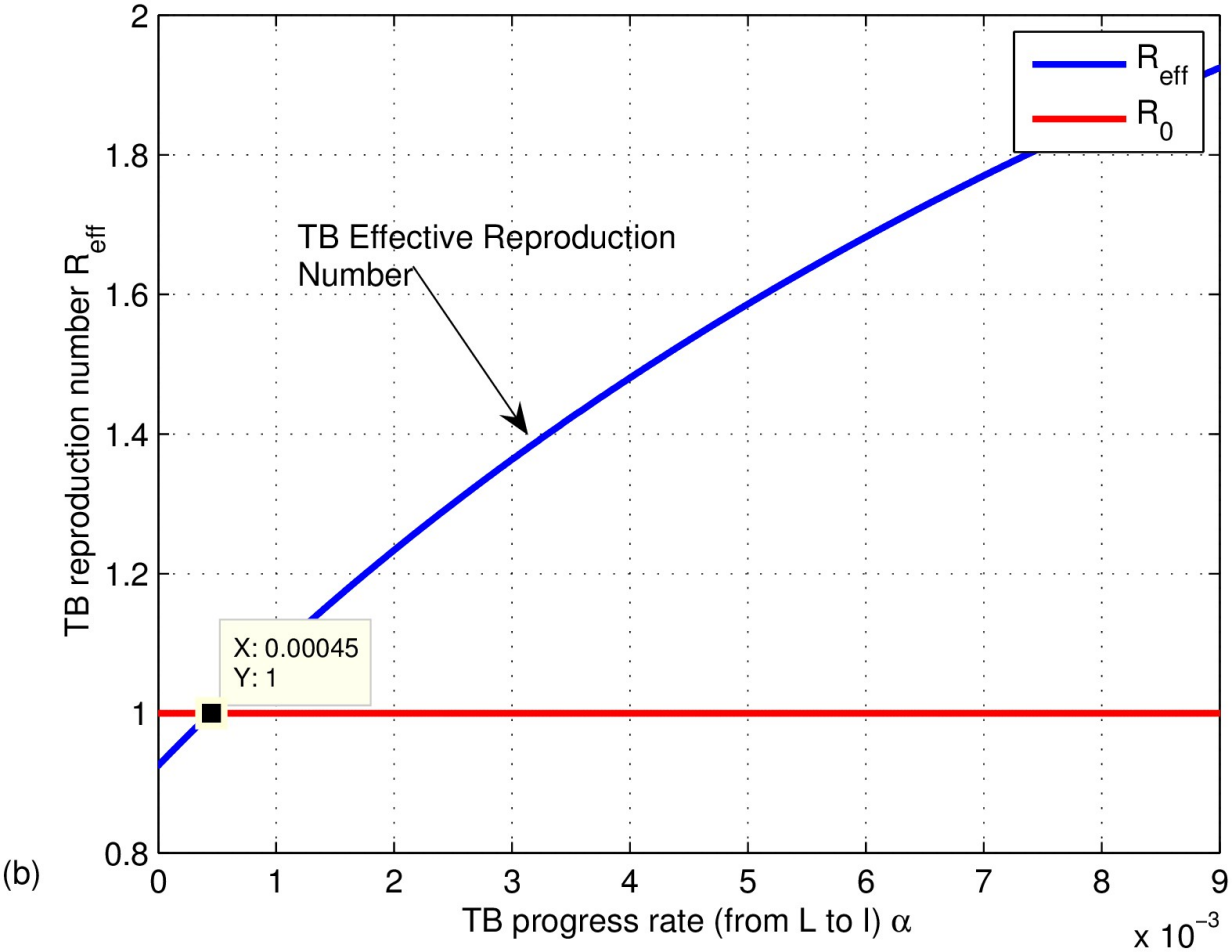
Effect of progress rate (*α*) on the effective reproduction number *R*_*eff*_.

**Fig 11:**
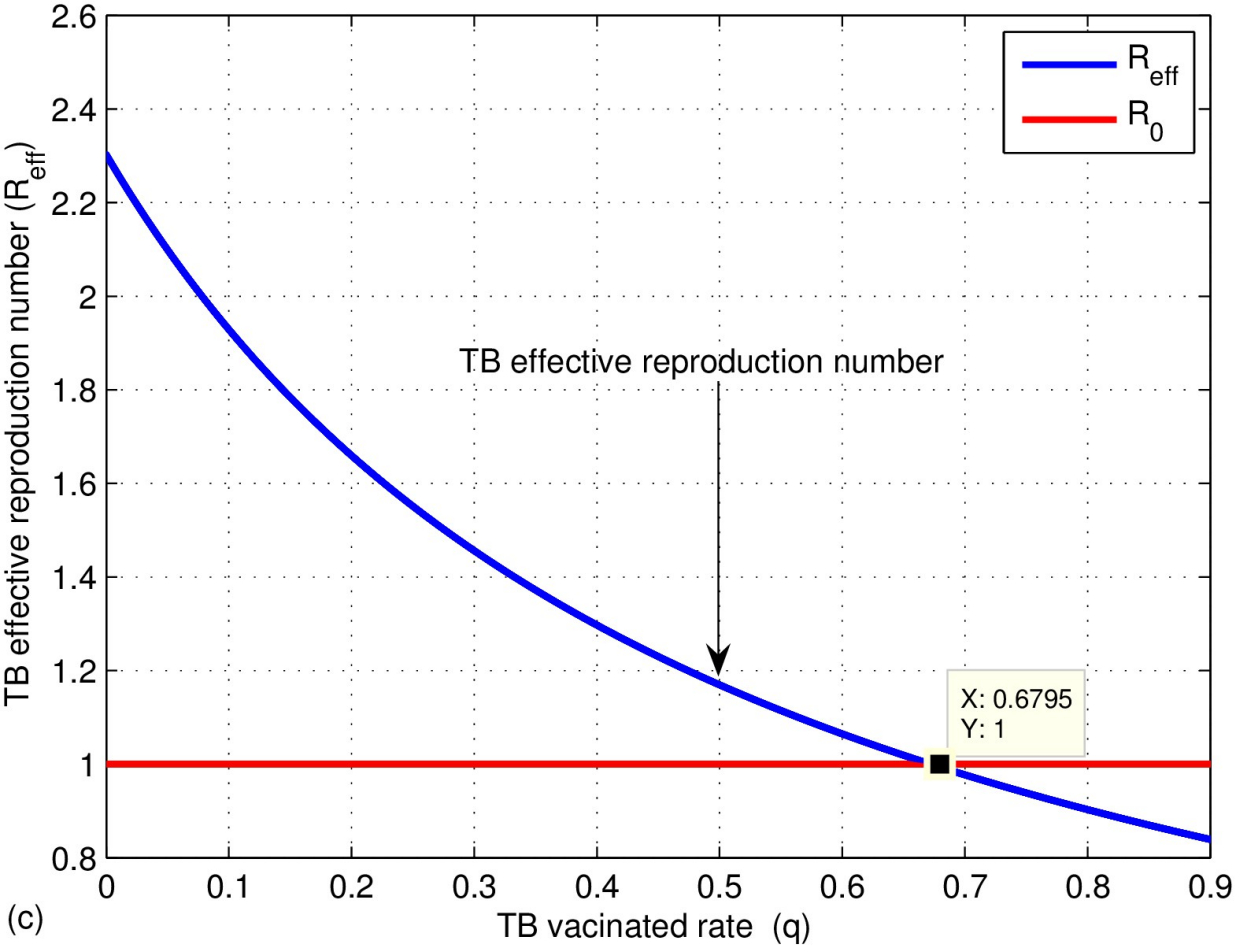
Effect of TB vaccinated rate (*q*) on the effective reproduction number *R*_*eff*_.

**Fig 12:**
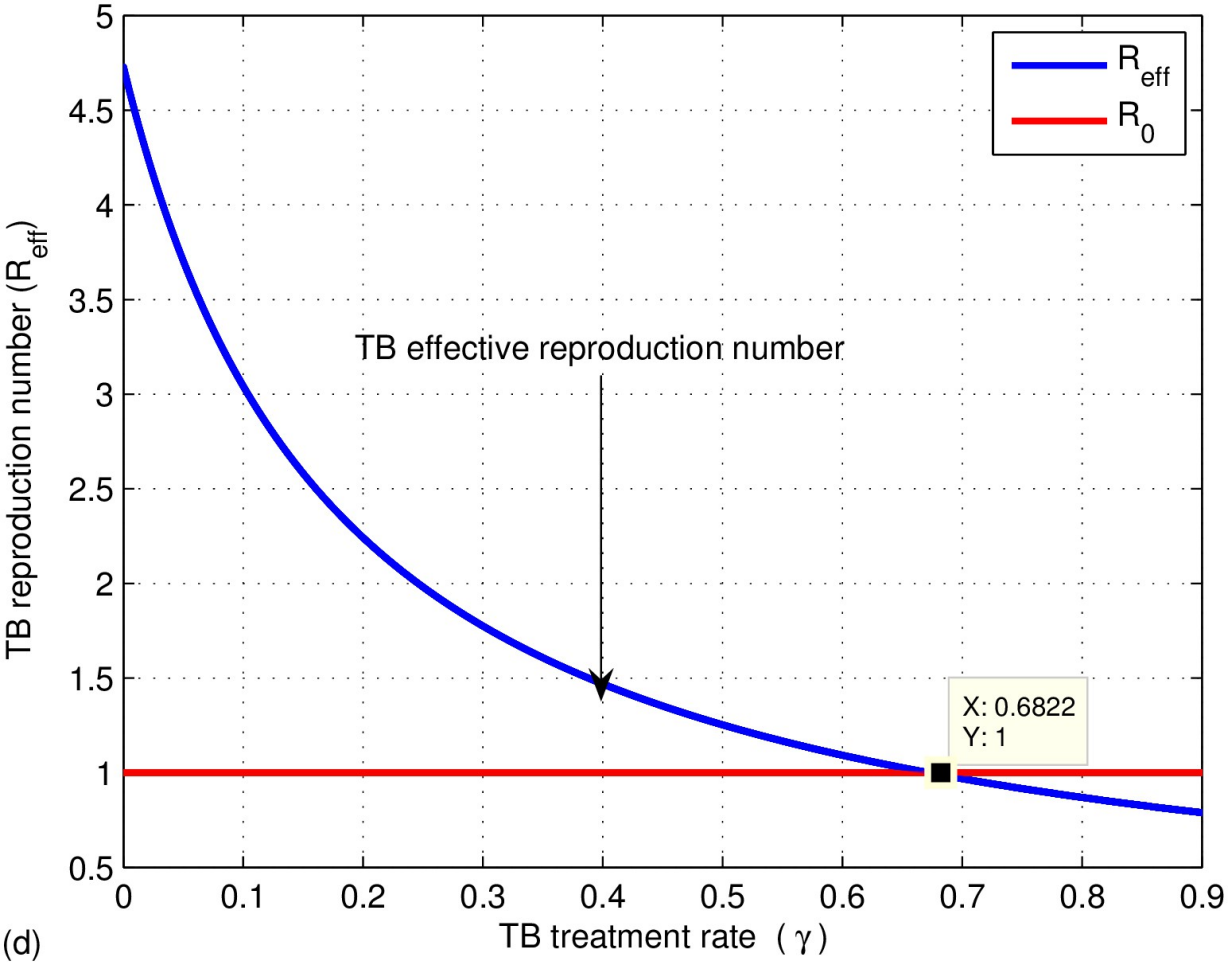
Effect of TB treatment rate (*γ*) on the effective reproduction number *R*_*eff*_.

**Fig 13:**
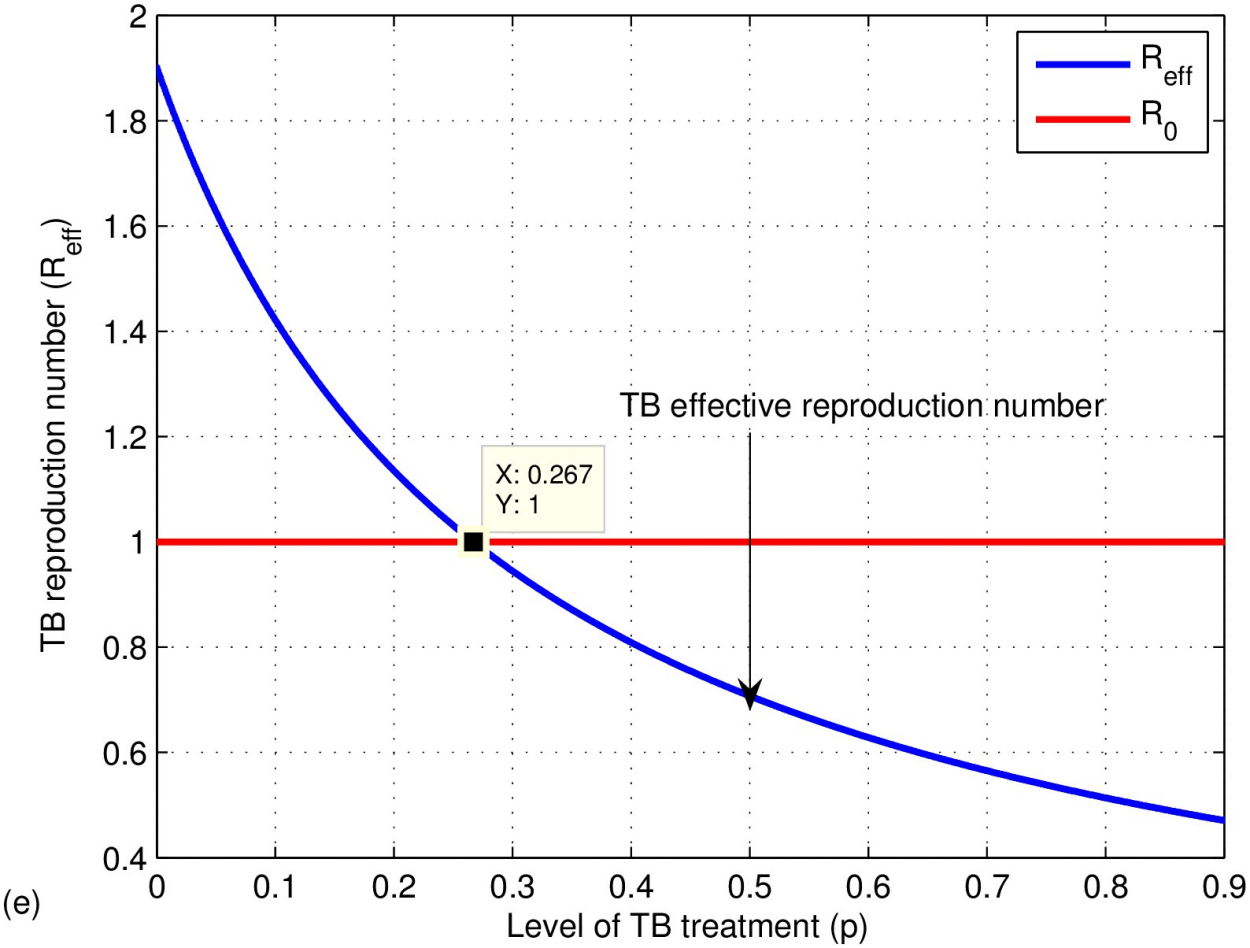
Effect of successful treatment rate (*p*) on the effective reproduction number *R*_*eff*_.

**Fig 14:**
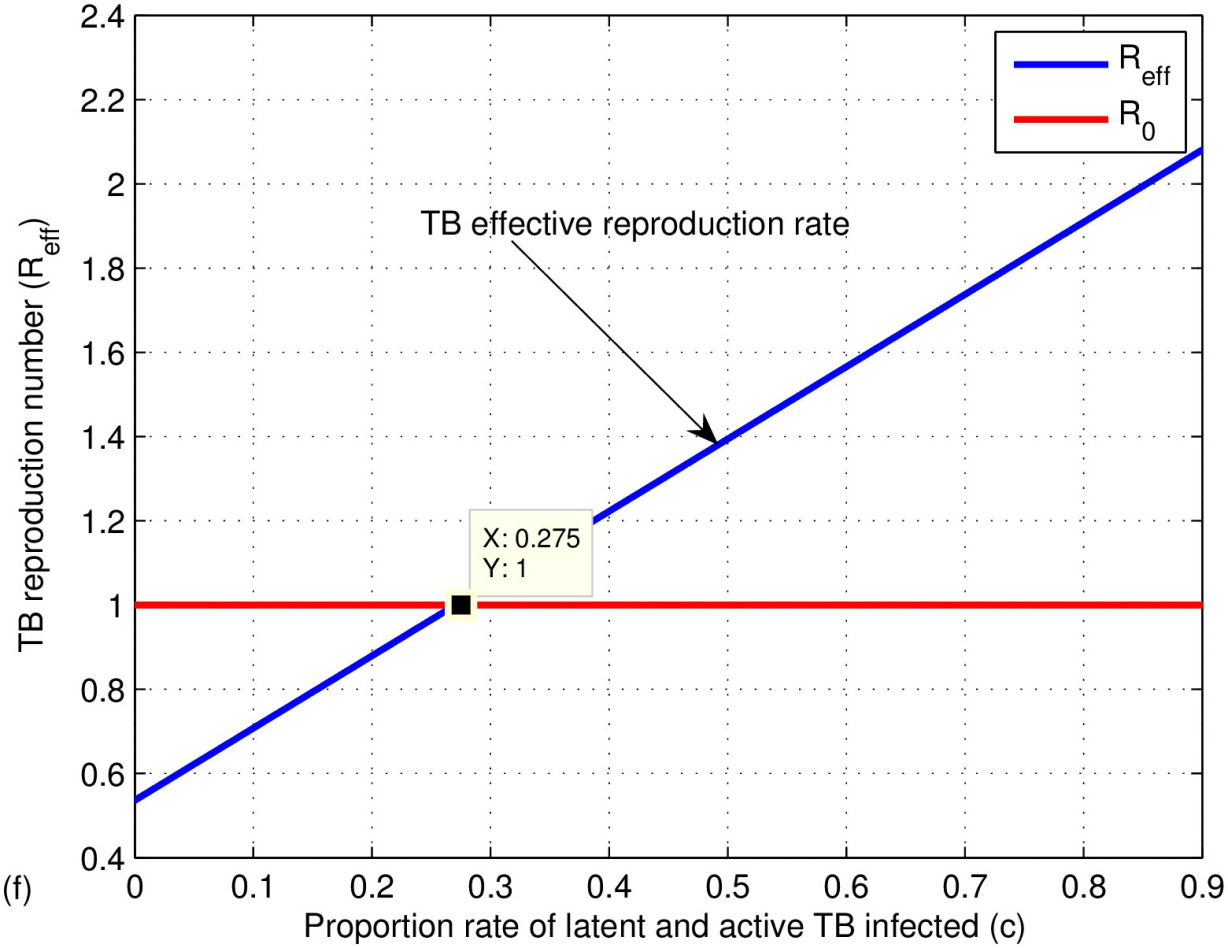
Effect of proportion rate (*c*) on the effective reproduction number (*R*_*eff*_).

The graphs in Figs 9–14 represent the effect of the model parameters on the effective reproduction number. From Figs 9–14, we can see the effect of each parameter on the effective reproduction number of this work. As shown in Fig 9, if the transmission contact rate before media alert (*β*_1_) is too small (< 0.00024), the reproduction number is reduced (below 1); otherwise, *R*_*eff*_ increases. This shows the fact that when (*β*_1_) increases, the TB disease infection will be increased. That’s why the basic reproduction number (*R*_0_) is used to describe the contagiousness or transmissibility of infectious agents. In epidemiology, the basic reproduction number (*R*_0_) is a term that describes the expected number of infections produced by a single case in a susceptible population. Fig 10 illustrates that a low progress rate (*α*) from latent to active TB results in a decreased reproduction number. Conversely, a high (*α*) leads to an increased reproduction number, suggesting that TB could spread more widely. To effectively eradicate TB, it is crucial to significantly lower the progress rate from latent to active infection. Fig 11 demonstrates that increasing the TB vaccine rate (*q*) leads to a decrease in the effective reproduction number (*R*_*eff*_). This indicates that the TB vaccine is effective in lowering (*R*_*eff*_), particularly when the vaccination rate exceeds 70% (*q* > 0.6795).

The effect of treatment is also shown in Fig 12, which shows a decrease in the effective TB reproduction number when taking more treatment adherence (> 0.6822) otherwise, it increases. Hence, to be effective in controlling TB, increase the undergoing treatment rate (*γ*). Fig 13 introduces the effect of successful treatment on the effective TB reproduction number. If the successful treatment is *p* > 0.267 then the effective TB reproduction number decreases, which is below one. If the successful treatment is *p* < 0.267 (it means more treatment failure happened), then the effective TB reproduction number increases, which is above one. As we can see from our results, if the successful treatment is high or *p* > 0.267, then the effective reproduction number of TB is reduced. If the treatment fails, the disease may progress and develop into drug-resistant TB disease. Therefore, in the next work, mathematical model analysis of drug-resistant TB should be done considering the first- and second-line treatment failure of TB disease. From Fig 14, the reproduction number of TB is reduced when the proportion rate (*c*) is less than 0.275 otherwise, it increases. It indicates if susceptible people are contracted by active TB above 27%, the effective reproduction number becomes high and the spread of TB increases.

**Fig 15:**
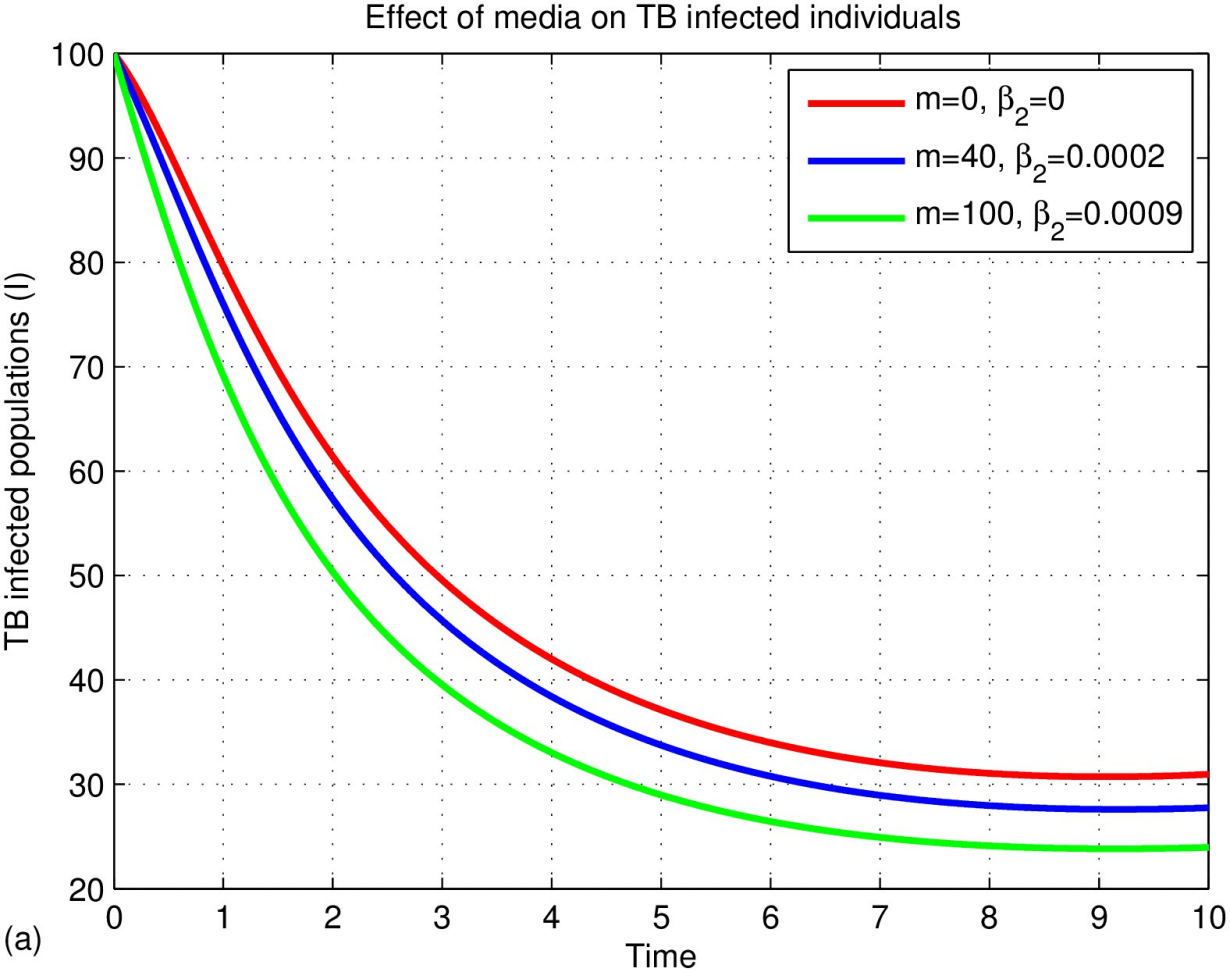
Effects of media on TB infected populations.

**Fig 16:**
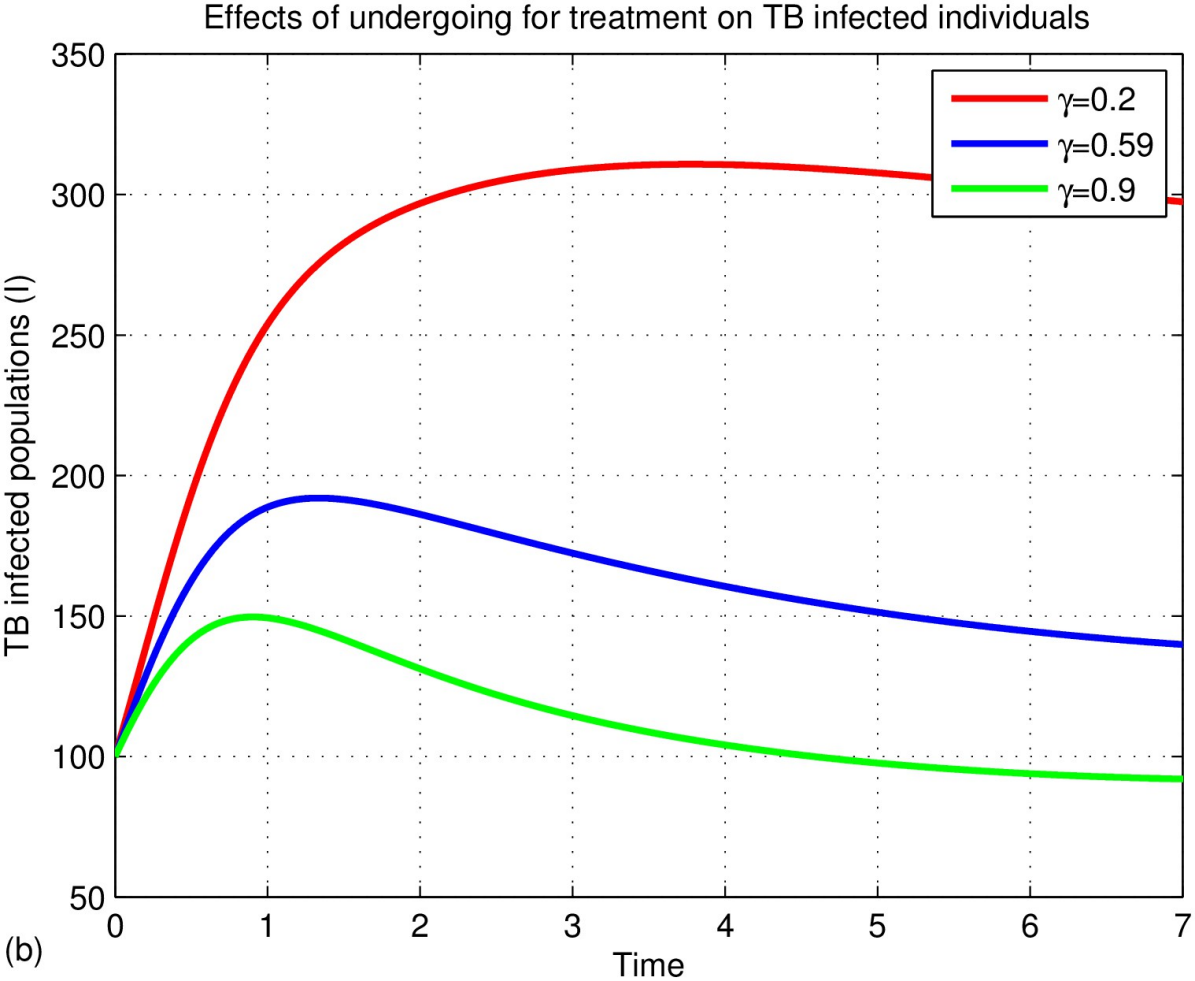
Effects of treatment on TB infected populations.

**Fig 17:**
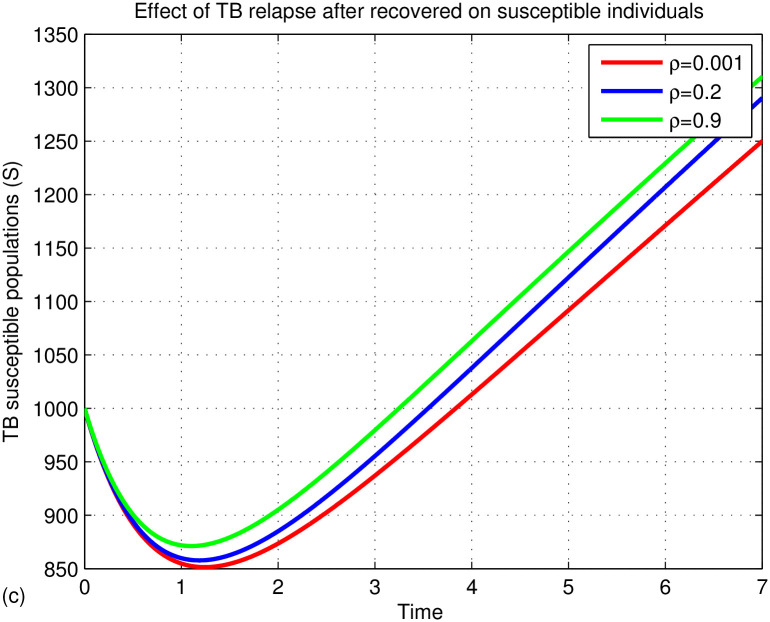
Effects of TB relapse on TB infected populations.

**Fig 18:**
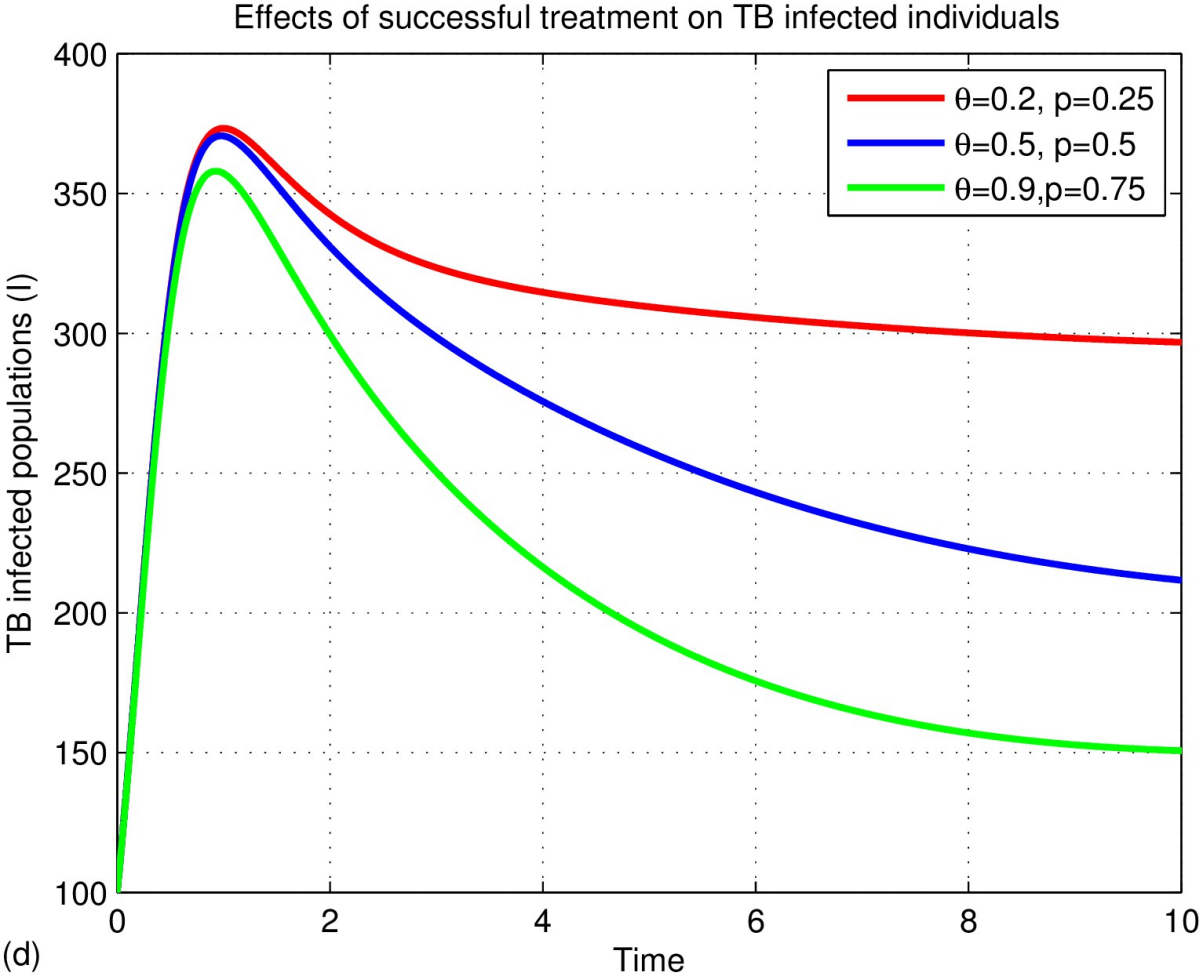
Effects of recovery rate on TB infected populations.

The graphs in Figs 15–18 represent the parameter effects on susceptible and TB infected populations. In Figs 15–18, we recognize the impact of the parameters on TB disease. Fig 15 indicates the impact of media on TB-infected populations compared with and without media efficacy. We have observed that TB disease is reduced in the presence of media compared to its absence. As the transmission rate *β*_2_ after media alert increases, the TB infected population decreases. Hence, we conclude that media has positive role in reducing the TB disease. Media are essential tools for communication, playing a critical role in cultural and societal development. Their effectiveness varies across different cultures and contexts. In multicultural and rural areas like Ethiopia, media outreach is most effective when aligned with local traditions and customs. For instance, health professionals can utilize community gatherings to raise awareness about tuberculosis (TB) by discussing its transmission and severity. Distributing informative leaflets during local meetings can further enhance outreach and help reduce TB spread. Additionally, it’s important to explore optimal methods for delivering TB information through radio and television to ensure community engagement and awareness. Fig 16 illustrates the impact of treatment on TB disease, showing that a small number of treated individuals has little effect on reducing infections, while significant reductions occur when treatment rates exceed 90%. Fig 17 highlights the effects of TB relapse, indicating that increased relapses lead to a larger susceptible population, whereas fewer relapses reduce this impact. Fig 18 examines successful treatment versus treatment failure, revealing that a low recovery rate results in treatment failure and a limited decrease in infections. In contrast, a high recovery rate leads to more successful treatments, reducing TB cases. Overall, increased successful treatment correlates with a higher recovery rate and a decrease in the TB-infected population, while high treatment failure contributes to rising infection rates.

## 7 Conclusion and recommendation

In this paper, a nonlinear deterministic mathematical model of TB transmission dynamics has been developed. We have demonstrated the well-posedness properties of the model’s solutions within a biologically feasible region. Additionally, we computed both the disease-free and endemic equilibrium points of the model and analyzed the local stability at these points concerning the effective reproduction number (*R*_*eff*_). We proved that the disease-free equilibrium points are stable when the effective reproduction number is less than one. The endemic equilibrium points exist, and a backward bifurcation also occurs, indicating that *R*_*eff*_ < 1 is a necessary but not sufficient condition for eradicating TB from the community. We also calculated both the basic and effective reproduction number of the model. The optimal control problem was constructed by considering two controls: *u*_1_, representing TB prevention, and *u*_2_, representing TB treatment control. From the numerical simulation results, we found that the combination of both control strategies (*u*_1_ & *u*_2_) is the most effective approach, while *u*_1_ and *u*_2_ alone serve as secondary and tertiary strategies for controlling TB transmission.

Furthermore, we discussed the impact of media coverage, which plays a significant role in controlling TB, as illustrated in Fig 15. The numerical simulations indicate that the TB vaccine is effective in reducing the effective reproduction number. Moreover, high treatment failure correlates with a low recovery rate, leading to an increase in the infected population. Conversely, higher successful treatment rates result in an increased recovery rate, thereby decreasing TB cases. Based on this analysis, we recommend that future work focus on reducing the rate of transition from latent TB to active TB, as this could significantly enhance TB prevention efforts. Additionally, we plan to study the transmission dynamics of drug-resistant TB strains with media influence and incorporate real data in my future research.

### 7.1 Limitations of the study

The limitation of this work is the lack of accurate collected data and not properly setting the cost-effective analysis of the control parameters when designing the control units. Also, we did not rank, analyze, or differentiate specific media to predict media effectiveness.
